# Contrasting Cannabinoid Receptor 2 (CB2R)-Associated Responses in Two Different Cerebrovascular Endothelial Cell Lines in a Blood Brain Barrier Model in the Context of HIV

**DOI:** 10.3390/cells15201889

**Published:** 2026-10-16

**Authors:** Violaine Delorme-Walker, Kaylin Au, Wei Ling Lim, Takayo Sasaki, Tomomi Furihata, Daniel de Siqueira Lima, Nydia Camille Grubbs, Jenifer Castaneda, Irina Kufareva, Jennifer Iudicello, Richard Milner, Maria Cecilia Garibaldi Marcondes

**Affiliations:** 1 San Diego Biomedical Research Institute, San Diego, CA 92121, USA; 2 Laboratory of Advanced Drug Development Sciences, School of Pharmacy, Tokyo University of Pharmacy and Life Sciences, Tokyo 192-0392, Japan; 3 HIV Neurobehavioral Research Center, University of California, San Diego, CA 92103, USA; jiudicello@health.ucsd.edu; 4 Honors Biomedical Science Program, University City High School, San Diego, CA 92122, USA; 5 Skaggs School of Pharmacy and Pharmaceutical Sciences, University of California, San Diego, CA 92093, USA; 6 Department of Psychiatry, University of California, San Diego, CA 92037, USA

**Keywords:** cannabis, human immunodeficiency virus, blood-brain barrier, latency

## Abstract

Despite the success of suppressive antiretrovirals, infection with human immunodeficiency virus (HIV) is associated with several comorbidities which include negative consequences to the central nervous system (CNS), where disruption of the blood-brain barrier (BBB) is a major underlying factor in the resulting chronic inflammation and contribution to the pathogenesis of neuroHIV. Currently, the use of cannabis and cannabinoid derivatives among persons living with HIV (PWH) is common. Despite perceived benefits, we have previously identified context-dependent effects of cannabis use, including its impact on vascular biomarkers. In this study, we used an in vitro polycellular BBB model with two different human cerebrovascular endothelial cell lines (hCMEC/D3 and HBMEC/ci18) to test the effects of cannabinoids and their receptors on barrier integrity and function in the context of the latent HIV brain environment, mimicked by the addition of conditioned media from HIV latently infected promonocytes. We found that the two brain endothelial cell lines had similar responses to HIV-conditioned media by increasing permeability to dextran and decreased expression of tight junction proteins. However, their response to cannabinoids, particularly via the cannabinoid receptor 2 (CB2R), was markedly different, with hCMEC/D3 cells showing improvement of BBB integrity by all measures, but HBMEC/ci18 cells showing no benefits or aggravation of damage. While the contrasting effects were not due to differences in cell viability or proliferation, differences CB2R-elicited baseline G protein-coupled receptor (GPCR) response thresholds between the two cell lines were significant, revealed by forskolin-induced cAMP levels that were 50–200-fold higher in hCMEC/D3 cells in comparison to HBMEC/ci18. This was correlated to higher baseline CB2R expression in hCMEC/D3, and was not an HIV-induced signaling defect in HBMEC/ci18. The CBD/THC-induced phosphorylation of p38 MAPK was also significantly stronger in hCMEC/D3 compared to HBMEC/ci18, was abrogated by a CB2R inverse agonist/antagonist. Motivated by the need to identify predictors of positive response to cannabinoids, our study suggests that CB2R levels and GPCR activation thresholds in cerebrovascular endothelium may be important candidate mechanistic biomarkers to be tested in primary cells, animal models, and longitudinal clinical cohorts, with the goal of distinguishing beneficial versus aggravating effects of cannabis to the BBB, particularly in the context of HIV.

## 1. Introduction

The management and cure of chronic HIV-1 infection remain a challenge due to the persistence of cellular reservoirs and chronic inflammation, despite antiretroviral therapies (ART) [1,2,3,4]. In the brain, where most HIV-1 targets and reservoirs are of myeloid origin [5,6,7], chronic inflammation contributes to neurological and neurocognitive impairment (NCI) [8,9,10,11,12]. One of the most overlooked consequences of chronic neuroinflammation is vascular injury, which is a risk factor for atherosclerosis and cardiovascular disease (CVD), but also a critical component of cerebrovascular pathology that is linked to NCI [13] especially in persons living with HIV (PWH) [14]. Substance use is also a prevalent HIV comorbidity [15,16,17] with effects on both CVD and NCI. The mechanisms by which addictive substances and HIV interact are multifactorial and not well understood, with impacts going beyond reward and neurotransmitters [18,19]. Cerebrovascular endothelial cells and other components of the blood-brain barrier (BBB), as well as myeloid cells that are targets of HIV in the brain such as macrophages and microglia, all express receptors to neurotransmitters and drug compounds [20,21]. Thus, PWH that are substance users may show a wide range of cellular responses [22,23,24] that could depend on the type of drugs used and patterns of use [25].

Among used substances, cannabis is more prevalent among PWH than in the general population. Cannabis is used in many forms, both medically and recreationally [26,27]. Reported benefits include HIV symptom and treatment side-effect relief [28,29,30]. Observational studies report mixed effects of cannabis on disease characteristics such as CD4^+^ T-cells and HIV RNA [31,32]. Reported objective benefits of cannabis use in PWH include lower pro-inflammatory biomarkers in cells, plasma and cerebrospinal fluid (CSF) [33,34,35], lower immune activation and fewer CD16^+^ monocytes [34,36], with improved prognosis [37,38]. However, other studies have suggested that chronic cannabis use is linked to vascular disease [39,40] and increased risk of cardiovascular complications [41]. One study on chronic heavy smokers has shown more CVD events in both PWH and persons without HIV [42], suggesting that smoking may contribute to the outcome. Therefore, it is possible that the beneficial effects of cannabis on reducing inflammation may be counterbalanced by adverse vascular effects resulting from the mode of use.

Cannabis effects on inflammation, cognition, sleep, appetite, pain, and mood are thought to be mediated by the two primary constituents, (Δ9-tetrahydrocannabinol [THC] and cannabidiol [CBD], and their receptors (CBRs), along with the key enzymes (FAAH, MAGL) in the endocannabinoid system (ECS) [43]. These factors also modify endothelial states associated with vascular integrity and disorders [44,45,46,47,48,49] that influence BBB and have implications for brain health and NCI. Among the CBRs, CB1R and CB2R, as well as the orphan receptor GPR55, are known to be expressed by endothelial cells [50,51]. Both major cannabinoid receptors are Gi/o protein-coupled receptors (GPCR) that inhibit adenylyl cyclase activity, activate voltage-gated calcium channels, initiate mitogen-activated protein kinase (MAPK) and activate phosphoinositide 3-kinase (PI3K)-Akt pathways via induction of cyclic adenosine 3,5 monophosphate (cAMP) [52,53,54]. CB2R is of particular interest as it is broadly expressed, with known downstream effects of its signaling including regulation of cell growth and survival, endothelial cell functions, and modulation of immune cells that cause inflammation [55].

Recently, we found that moderate cannabis use may improve NCI [26] and prevent changes in the expression of polygenic components of vascular and leukocyte migration pathways in the context of HIV, but not in uninfected subjects [56]. Cannabis use patterns are linked to differences in the plasma levels of soluble vascular cell adhesion molecule (VCAM)-1, intercellular adhesion molecule (ICAM)-1 and urokinase activator plasminogen receptor (uPAR), that serve as biomarkers to detect and monitor vascular inflammation and NCI in the context of HIV [57,58,59,60]. However, other studies report no differences, or detrimental effects in HIV-negative populations [41], raising the possibility that the observed effects of cannabis, whether beneficial or detrimental, may be largely domain- and context-dependent.

Differential consequences of CB2R activation may also derive from genetic variations resulting in molecular and outcome differences [61,62], including in inflammatory and autoimmune pathogeneses [63]. Levels of CB2R on the surface of peripheral blood leukocytes may also serve as biomarkers of mental health disorders, such as schizophrenia [64], with a negative correlation between mRNA levels and processing or working memory speed [65].

The identification of CB2R on cerebral endothelial cells suggests that this receptor may play a role in regulating the BBB in correlation with inflammatory processes in the CNS. For instance, CB2R expression is highly upregulated on isolated blood vessels following brain injury, with a positive impact of its activation for recovery [55,66]. While in the human population, and especially in the context of HIV infection, overall benefits may be context-dependent [56], it is currently unknown whether activation of cannabinoid receptors on the BBB can directly benefit vascular health and thereby reduce damage occurring in the HIV-infected brain, considering potential individual differences in the expression of cannabinoid receptors.

With this question in mind, here we describe in vitro experiments that seeded from the observation that different cerebrovascular endothelial cell lines respond in opposite manners to exposure to agonists of cannabinoid receptor, particularly CB2R, in the context of HIV. By potentially mimicking different vascular responses to cannabinoids in humans, the comparison of two human cerebrovascular cell lines using an in vitro system facilitated the exploration of molecular mechanisms that may explain context-dependent effects or individual differences in vascular outcomes of cannabis, which influence BBB functions underlying inflammation in the brain. Our findings indicate that cannabinoid receptor signaling contributes to both beneficial and harmful effects that could explain context-dependent outcomes in PWH who use cannabis.

## 2. Material and Methods

### 2.1. BBB Cell Cultures

*Human Cerebrovascular Endothelial cells, Astrocytes and Pericytes*: The immortalized hCMEC/D3 cell line was donated by Dr. Vadivel Ganapathy (Texas Tech University Health Sciences Center). hCMEC/D3 cells (passage 14–24) were seeded on 0.1% gelatin-coated (Embryomax, Millipore, Burlington, MA, USA) culture dish in EndoGRO basal medium with EndoGRO-MV-VEGF supplement kit (Sigma-Aldrich, St. Louis, MO, USA) consisting of 5 ng/mL rhVEGF, 5 ng/mL rhEGF, 5 ng/mL rhFGF, 15 ng/mL rhIGF-1, 10 mM Glutamine, 1 µg/mL Hydrocortisone hemisuccinate, 0.75 U/mL Heparin sulfate, 50 μg/mL Ascorbic acid and 5% FBS (Millipore). Cells were maintained at 37 °C in a humidified atmosphere (5% CO_2_/95% air). Medium was changed every 3–4 days until cells reached confluence. Conditionally immortalized human brain microvascular endothelial cells clone 18 (HBMEC/ci18), human brain pericytes clone 37 (HBPC/ci37), and human astrocytes clone 35 (HASTR/ci35) were established, validated and kindly donated by Pr. Tomomi Furihata (Tokyo University of Pharmacy and Life Sciences, Tokyo, Japan). All cells were seeded on culture dishes coated with 50 μg/mL Cultrex 3-D Culture matrix rat Collagen I (R&D Systems, Minneapolis, MN, USA). For maintenance, HBMEC/ci18 cultures were grown in the same medium as for hCMEC/D3. HBPC/ci37 cells were maintained in complete pericyte growth medium (PGM) consisting of basal medium, 2% FBS, 1% pericyte growth supplement and 1% penicillin/streptomycin (ScienCell, Carlsbad, CA, USA). HASTR/ci35 cells were grown in complete astrocyte growth medium (AGM) consisting of DMEM + GlutaMAX, 10% FBS and 1% N2-supplement (Gibco, ThermoFisher Scientific, Waltham, MA, USA). All culture media for the conditionally immortalized human cells contained 4 μg/mL blasticidin S (Tocris, Minneapolis, MN, USA). HBMEC/ci18, HASTR/ci35 and HBPC/ci37 cell lines were maintained at 33 °C for growth and at 37 °C for differentiation, in a humidified atmosphere (5% CO_2_/95% air). Medium was changed every 3–4 days until cells reached confluence.

*Human promonocytes*: Human promonocytes uninfected (U937) and U937-derived latently infected with HIV-1 (U1) were maintained using RPMI-1640 medium (Gibco, Thermofisher, Waltham, MA, USA) supplemented with 10% fetal bovine serum (Hyclone, Cytiva, Marlborough, MA, USA), 2 mM Glutamine (Gibco) and 100 U/ml penicillin/streptomycin (Gibco). Cultures were maintained in 75 cm^2^ flasks (Genesee Scientific, San Diego, CA, USA) at 37 °C in a humidified atmosphere (5% CO_2_/95% air).

### 2.2. BBB Co-Culture System

Transwell inserts (clear polyester (PET) membranes, 6.5 mm diameter, 0.4 μm pores, #3470, Corning, Corning, NY, USA) in 24-well plates were coated with a combination of 50 µg/mL Cultrex 3D culture matrix rat collagen I (R&D systems, Minneapolis, MN, USA), 50 µg/mL Human Collagen IV (Advanced BioMatrix # 5022, Carlsbad, CA, USA) and 50 µg/mL fibronectin (#F1141, Sigma Aldrich, St. Louis, MO, USA) for 2 h at 37 °C. HBPC/ci37 pericytes were seeded on the underside of the transwells at a density of 2.7 × 10^4^ cells/cm^2^. HASTR/ci35 astrocytes were seeded on the bottom of the 24-well plate at a density of 2.5 × 10^4^ cells/cm^2^. After 24 h, the pericyte and astrocyte media were replaced with corresponding media without FBS and blasticidin S, with astrocyte medium also supplemented with 1 mM dibutyryl-cAMP (Selleckchem, Houston, TX, USA). Cells were allowed to differentiate for 24 h at 37 °C. Endothelial cells were then seeded on the apical side of the transwell at 10^5^ cells/cm^2^ or 1.3 × 10^5^ cells/cm^2^ for hCMEC/D3 and HBMEC/ci18, respectively. The transwell inserts with endothelial cells and pericytes were transferred into the 24-well plates containing the astrocytes. Cells were grown in EndoGRO culture medium mentioned above, Vascular endothelial growth factor (VEGF) and endothelial growth factor (EGF) free, in the apical chamber, and Neurobasal medium (Life Technologies, Carlsbad, CA, USA) containing 1% N2-supplement (ThermoFisher Scientific, Waltham, MA, USA) and 2 mM Glutamine (Gibco, Waltham, MA, USA) in the lower chamber. To control the establishment of a functional BBB, each endothelial cell line, hCMEC/D3 and HBMEC/ci18, was also seeded as monoculture in transwells. BBB co-cultures or endothelial cell monocultures were grown for 5 days. Endothelial monolayer integrity was checked by permeability measurements on day 5 and then on day 6 after exposure to conditioned medium or treatments. In each experiment, one transwell was used without cells as control for insert permeability.

### 2.3. HIV Conditioned Medium and P24 Levels

U937 and U1 cells were cultivated at the density of 10^6^ cells/ml in 75 cm^2^ flasks in EndoGRO medium without VEGF and EGF. High levels of virus expression were induced in U1 cells by stimulation with latency reversal agents: 10 nM and 1 μM PEP005 (Tocris, Minneapolis, MN, USA), 1 μM iBET151 (Sigma Aldrich, Burlington, MA, USA) or 10 ng/mL and 100 ng/mL PMA (Sigma Aldrich, Burlington, MA, USA) for 48 h. As controls, U937 cells were subjected to the same treatments. Culture supernatants were harvested by centrifugation after 5 days (U937 and U1) or after 48 h in presence of latency reversal agents. Conditioned media were stored at −20 °C until used in assays. Levels of p24 antigen in U1 conditioned media were determined by ELISA (HIV-1 p24 ELISA assay, Xpress Bio, Frederick, MD, USA), according to the manufacturer’s instructions. The U1 conditioned medium used in the BBB assays was harvested after 5 days in culture and applied undiluted to the BBB co-cultures. p24 levels averaged 360 pg/ml (±42.86) across experiments. U937 media did not have detectable p24.

### 2.4. Vascular Inflammation and Network Analysis

To characterize the contents of the conditioned media, U937 and U1 supernatants were analyzed using LEGENDplex^TM^ Human Vascular Inflammation (BioLegend, San Diego, CA, USA) multiplex bead-based assay Panels 1 and 2, following the manufacturer’s instructions. After completion of the reaction, the samples were acquired on a CytoFLEX S flow cytometer (Beckman Coulter, Brea, CA, USA) and the results were analyzed using LEGENDplex™ Data Analysis Software Version 2025-05-01 (BioLegend). The concentration of each analyte was determined using a standard curve generated in the same assay. All conditions were tested in triplicate, and the assays were repeated at least four times. Network analysis and visualization was performed using Cytoscape v3.10.2 software [56,67,68] with ReactomeFIPlugIn [69].

### 2.5. Treatments

Endothelial cell monolayers seeded on transwell inserts, glass coverslips or cell culture plates for 5 days were exposed to control conditioned medium (U937) or HIV-conditioned medium (U1) for 24 h at 37 °C for hCMEC/D3 or 33 °C for HBMEC/ci18. Cells were pre-treated with DMSO (0.1% *v*/*v*) (ATCC, Frederick, MD, USA) as control, or with 0.1 μM, 1 μM or 10 μM cannabinoid receptor agonists or antagonists, respectively, as follow: ACEA and AVE1625 for CB1R, HU-308 and AM630 for CB2R, O-1602 and ML 193 for GPR55, respectively (all from Tocris, Minneapolis, MN, USA). Cells were pre-treated for 30 min in EndoGRO VEGF, EGF-free medium with DMSO (0.1% *v*/*v*), cannabinoid receptor agonists or antagonists, prior to 24 h incubation with conditioned medium supplemented with the same compounds.

Endothelial monolayers were also incubated for 24 h with 0.01 μM, 0.1 μM, 1 μM or 10 μM Cannabidiol (CBD, PhytoLab, Millipore Sigma, Burlington, MA, USA) or (−)-trans-∆^9^-tetrahydrocannabinol (THC, RTI International, Research Triangle Park, NC, USA), alone or together, in combination with control conditioned medium (U937) or HIV-conditioned medium (U1). Methanol and ethanol at respective dilutions were used as vehicle controls to THC and CBD, respectively.

### 2.6. Measurement of Paracellular Permeability

Effects of HIV-conditioned media and CB2R signaling on BBB integrity were assessed by measuring paracellular permeability to three fluorescently labeled dextrans: 4 kDa FITC-conjugated dextran (Millipore Sigma), 10 kDa Cascade Blue-conjugated dextran (Invitrogen, Waltham, MA, USA) and 40 kDa Texas Red-conjugated dextran (Invitrogen). Briefly, hCMEC/D3 and HBMEC/ci18-containing inserts were washed with Hank’s balanced salt solution (HBSS) containing 1.26 mM CaCl_2_ and 0.49 mM MgCl_2_ + 10 mM Hepes pH 7.5. Inserts were then incubated with 700 μL HBSS + 10 mM Hepes pH 7.5 in the basolateral side and 300 μL HBSS + 10 mM Hepes pH 7.5 containing 10 µg/mL of each fluorescently labeled dextran in the apical side. Inserts were incubated 1 h at 37 °C for hCMEC/D3 or 33 °C for HBMEC/ci18. The medium from the basolateral side was collected and fluorescence intensity was measured in triplicate on a VersaMax Spectrophotometer (Molecular Devices, San Jose, CA, USA) using the following wavelengths: Ex405/Em440 for the Cascade Blue-conjugated dextran, Ex485/Em525 for the FITC-conjugated dextran and Ex560/Em615 for the Texas Red-conjugated dextran. The concentration of the corresponding dextran particles was determined, and the permeability coefficient values were calculated using the following equation, as previously described [70,71,72]:

P = (Vr/C0) × (1/S) × (C1/t),

where P is the apparent permeability, Vr is the volume of medium in the basolateral side of the chamber (Vr = 0.7 cm^3^), C0 is the concentration of the fluorescent dextran in the apical side of the transwell at t0 (C0 = 10 μg/mL), S is the surface area of the monolayer (S = 0.33 cm^2^), C1 is the concentration of the fluorescent dextran in the basolateral side of the chamber after incubation and t is the incubation time (t = 3600 s).

### 2.7. Cell Viability

Endothelial cells were seeded at the density of 10^5^ cells/cm^2^ in 24-well plates coated with 0.1% gelatin (hCMEC/D3) or 50 μg/mL Cultrex 3-D Culture matrix rat Collagen I (HBMEC/ci18). Cells were cultured in EndoGRO medium without VEGF and EGF for 5 days before being incubated for 24 h in presence of conditioned media, or in cell culture medium with 0.1% DMSO (*v*/*v*), 0.1 μM, 1 μM or 10 μM HU-308. Each culture condition was tested in triplicate. Three wells without cells were used as blank control. Cell viability was measured with CyQUANT^TM^ XTT Cell Viability Assay (ThermoFisher Scientific), according to the manufacturer’s instructions. XTT reagent was incubated with the cells for 4 h at 37 °C or 33 °C for hCMEC/D3 and HBMEC/ci18 cells, respectively. Absorbances at 450 nm (XTT specific) and at 660 nm (background signal) were measured on a VersaMax Spectrophotometer. Absorbance was quantified as follows: Absorbance = [Abs 450 nm (sample) − Abs 450 nm (blank no cells)] − Abs 660 nm (sample). The final absorbance was normalized to U937 control conditioned medium for experiments with U1, or to cell culture medium with 0.1% DMSO for experiments with HU-308.

### 2.8. Cell Proliferation

hCMEC/D3 and HBMEC/ci18 cells were seeded at the density of 10^5^ cells/cm^2^ in 12-well plates containing glass coverslips (#1.5, Carolina, Burlington, NC, USA) pre-coated with 50 µg/mL Cultrex 3D culture matrix rat collagen I (R&D systems). Cells were cultured in EndoGRO medium without VEGF and EGF for 5 days before being incubated for 24 h in presence of conditioned media, with 0.1% DMSO (*v*/*v*) or 10 μM HU-308, in combination with 10 μM 5-Bromo-2′-deoxy-Uridine (BrdU). Immunofluorescence detection of BrdU was performed using BrdU labelling and detection kit I (Roche molecular biochemicals, Indianapolis, IN, USA), according to the manufacturer’s instructions. Cells were co-stained with DAPI to detect nuclei. Coverslips were mounted on slides with Prolong Gold Antifade (Invitrogen). Images were acquired using a 20x objective on an Axioskop2 plus microscope (Carl Zeiss, Dublin, CA, USA) equipped with an Infinity 3S camera (Lumenera, Ottawa, ON, Canada) and Infinity Analyze imaging software (Lumenera). Image analysis and quantification of the percentage of BrdU positive cells over total cells were performed with ImageJ/FIJI software (v.2025-05-14, NIH, Bethesda, MD, USA).

### 2.9. Detection of Forskolin-Induced Cyclic AMP

hCMEC/D3 and HBMEC/ci18 endothelial cells were seeded on a 96-well plate at 10^5^ cells/cm^2^ and cultured as above until forming a monolayer. Following 24 h of treatments as described above, the cells were incubated with cAMP assay buffer (HBSS 1x, 10 mM Hepes and 10 μM of Forskolin). Cyclic AMP was measured using the HitHunter cAMP assay for Biologics kit (DiscoverX Corporation, Fremont, CA, USA) following manufacturer’s protocols. Chemiluminescence signals were measured on a standard luminometer at 0.5 s/well. All conditions were tested in duplicate and assays were performed in independent triplicates.

### 2.10. RT-PCR

hCMEC/D3 and HBMEC/ci18 cells were seeded at the density of 1.5 × 10^5^ cells/well on 12-well plates coated with 0.1% gelatin (Millipore) or 50 µg/mL Cultrex 3D culture matrix rat collagen I, respectively. Cells were cultured in EndoGRO medium without VEGF and EGF for 5 days before being incubated for 24 h in presence of conditioned media, with 0.1% DMSO (*v*/*v*) or 10 μM HU-308. Total RNA was extracted from samples using Nucleospin RNA isolation kit (Macherey-Nagel, Allentown, PA, USA) and cDNA was obtained using RT^2^ First-strand kit (Qiagen, Germatown, MD, USA), according to the manufacturers’ instructions. SYBR Green real-time PCR was performed using the RT^2^ PCR Primer set for human CB1 receptor (CNR1, GenGlobe ID: PPH01504A-200, Qiagen), CB2 receptor (CNR2, GenGlobe ID: PPH02723A-200, Qiagen) and GPR55 (GenGlobe ID: PPH11293B-200, Qiagen) primers. Human GAPDH (GenGlobe ID: PPH00150F-200, Qiagen) was used as housekeeping control. The expression was normalized to mRNA level of the housekeeping gene GAPDH and to mRNA level in control conditioned medium (U937). The relative mRNA expression was determined by measuring 2^−ΔΔCt^ values.

### 2.11. Tight Junction Immunofluorescence and Imaging

After measurement of endothelial cell permeability, transwells were briefly washed three times in calcium- and magnesium-free phosphate buffered saline (PBS). Cells were immediately fixed in cold methanol/acetone (50%/50%) for 20 min at −20 °C. After rinsing in PBS, cells were incubated with a blocking and permeabilization solution consisting of PBS supplemented with 5% FBS and 0.3% Triton X-100. Endothelial cells on the apical side of the transwell were incubated with primary antibodies against Zonula Occludens-1 (ZO-1, Invitrogen #61-7300), Occludin (Invitrogen #33-1500) or CD31 (BD Pharmingen, San Diego, CA, USA #555025). Pericytes on the underside of the transwell membrane were incubated with antibodies against NG2 Chondroitin Sulfate (Sigma #AB5320). Astrocytes on the bottom of transwells were labeled with antibodies against Glial Fibrillary Acidic Protein (GFAP, Sigma #MAB360) or Glutamine Synthetase (GS, Sigma # G2781). All antibodies were diluted 1:100 in PBS supplemented with 2% BSA and 0.1% Tx-100. Transwells were incubated overnight at 4 °C in a humidified chamber. Following rinses in PBS, cells were incubated for 3 h with secondary antibodies donkey anti-rabbit AlexaFluor488 or anti-mouse AlexaFluor647 in combination with DAPI to stain nuclei. Insert membranes were cut out with a scalpel, mounted on a glass slide with Prolong Gold Antifade and coated with glass coverslip.

Fluorescent images were acquired either on an Axioskop2 plus microscope (Carl Zeiss, Dublin, CA, USA) equipped with an Infinity 3S camera (Lumenera) controlled by Infinity Analyze imaging software (Lumenera) by using a 40× objective or on a spinning disk (Yokogawa, Tokyo, Japan) Nikon TE 2000-U (Nikon, Melville, NY, USA) equipped with a CoolSnapHQ camera (Photometrics, Tucson, AZ, USA) using a 40×/1.3 NA Plan Fluor objective lens (Nikon). The intensity of the entire area of fluorescence images was considered for analysis. The percentage of cells with altered junctions was measured by counting the number of cells with discontinued ZO-1 labelling around the cell, over the total number of cells. All quantifications were performed using ImageJ/FIJI (NIH).

### 2.12. Western Blots

Endothelial cell monolayers were stimulated with 10 μM HU-308, 1 μM CBD + 1 μM THC, or 10 μM AM630 with 1 μM CBD + 1 μM THC for 5, 10, 15, 30 or 60 min. 0.1% DMSO (*v*/*v*) or 0.1% MeOH/EtOH were used as vehicle where appropriate. In CBD/THC conditions, cells were pretreated with 0.1% DMSO (*v*/*v*) or 10 μM AM630 for 30 min before adding CBD/THC alone or in combination with AM630. Cells were lysed in radio-immunoprecipitation assay buffer (RIPA—Thermo Fisher Scientific, Waltham, MA, USA) in the presence of Complete protease inhibitor cocktail tablets (Roche Molecular Biochemicals, Indianapolis, IN, USA) and Phosphatase Inhibitor Cocktails II and III (MedChemExpress, Monmouth Junction, NJ, USA). Protein concentration was measured using a Pierce™ BCA Protein Assay Kit (Thermofisher). Protein was stored in −20 °C until use. Forty micrograms of protein were loaded into lanes of SDS-PAGE electrophoresis (BioRad, Hercules, CA, USA) in 4–20% gradient gels under reducing conditions. Transfer and immunodetection were performed as described [73]. Non-specific antibody binding was blocked using 5% Bovine Serum Albumin Fraction V (BSA, Genesee Scientific, San Diego, CA, USA) for 1 h at room temperature. Immunoblotting was carried out with antibodies against total P38 MAPK mouse (ThermoFisher Scientific), phosphor-P38 MAPK (Thr180/Tyr182) rabbit (pp38, Cell Signaling, Danvers, MA, USA), and Hsp90 (Cell Signaling, Danvers, MA, USA), followed by secondary antibody HRP-conjugated anti-rabbit IgG or anti-mouse (both from Millipore Sigma, Burlington, MA, USA). Blots were developed with 1:1 solution of Super Signal West Pico Chemiluminescent Substrate and Luminol/Enhancer (Thermo-Fisher Scientific, Rockford, IL, USA), and bands were scanned on a ChemiDoc XRS+ imaging system (BioRad). Intensities were calculated in Fiji/ImageJ (National Institute of Health, Bethesda, MD, USA). Experimental bands were normalized to the intensity of Hsp90 bands in each sample.

### 2.13. Statistical Analysis

Data are expressed as mean ± standard error of mean (SEM). Each experiment was performed in triplicate. Unpaired, two-tailed Student’s *t*-test was used to evaluate significant differences between two groups. Analysis of Variance followed by Bonferroni’s multiple comparisons were used for condition and cell line comparisons when applicable. *p* values < 0.05 were considered statistically significant. Analysis and graphs were made using GraphPad Prism version 10.6.1 (GraphPad Software, Boston, MA, USA).

## 3. Results

**Figure 1:**
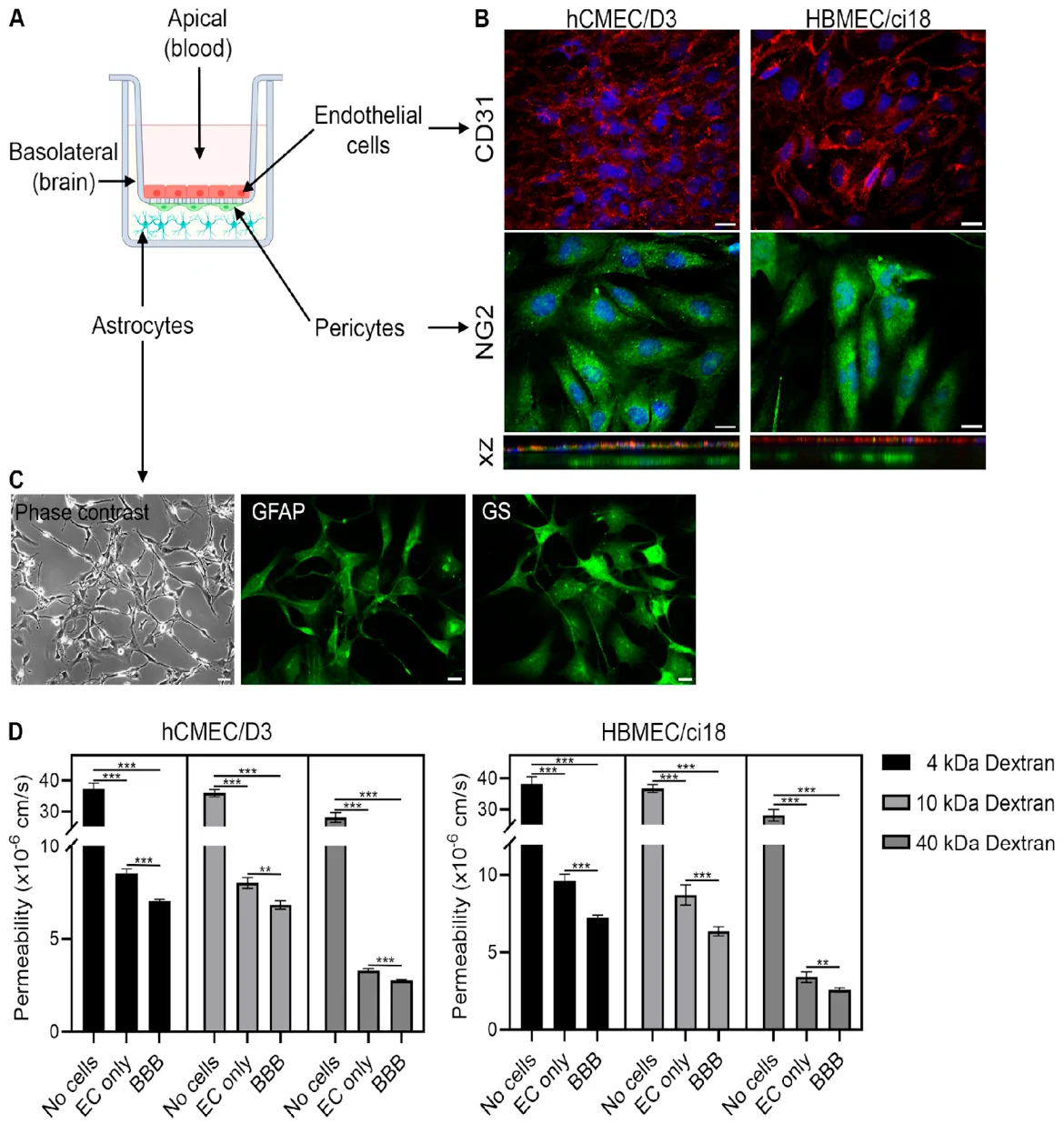
Construction of a polyculture human BBB system. (**A**) Scheme of the human BBB model containing endothelial cells (hCMEC/D3 or HBMEC/ci18), and supporting cells, astrocytes (HASTR/ci35) and pericytes (HBPC/ci37), and indication of the blood and brain compartments. (**B**) Immunofluorescence indicating CD31+ endothelial cell monolayers and NG2+ pericytes. Scale bar, 20 µm. Bottom panels are z projections showing the 3D coexistence and interaction between cellular types in the model. (**C**) Detection of astrocytes in the basolateral compartment, visualized by phase contrast and confirmed by immunostaining for glial fibrillary acidic protein (GFAP) and glutamine synthase (GS). Scale bar, 20 µm. (**D**) Permeability to dextran particles (4, 10 and 40 kDa) in membranes with no cells (control), with endothelial cells alone (EC only, hCMEC/D3 or HBMEC/ci18), and with the complete multicellular BBB system. N = 5 biological replicates. Statistical significance is indicated by lines. ** *p* < 0.01, *** *p* < 0.0001, ANOVA followed by Bonferroni’s posthoc test.

We have developed an in vitro BBB model (Figure 1A) using cerebrovascular endothelial cells (CD31-positive), pericytes (NG2-positive, Figure 1B) and astrocytes (GFAP-positive, Figure 1C), which form effective physical interactions detectable on Z-stack images and establish a functionally tight barrier to large molecules such as dextran (4, 10 and 40 kDa), especially when compared to endothelial cell (EC) monocultures (Figure 1D). The establishment of functional BBBs was successful using either one of two different cerebrovascular endothelial cell lines, hCMEC/D3 and HBMEC/ci18 (Figure 1D).

**Figure 2:**
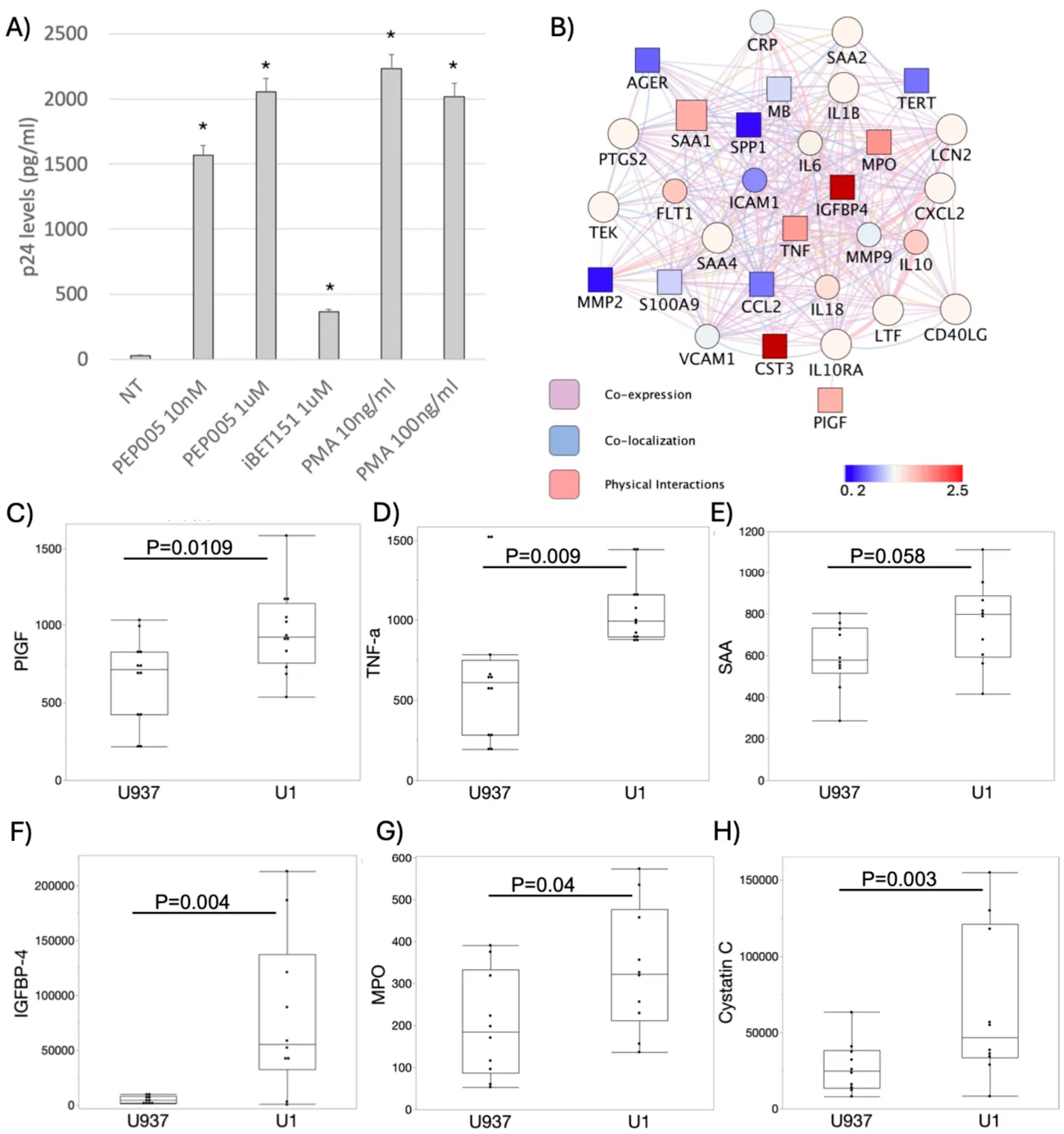
Confirmation of latency and characterization of the conditioned media. (**A**) Levels of p24 measured by ELISA in supernatants from U1 cells that were not treated (NT), or treated with latency reversal agents PEP005, iBET151 and PMA at indicated concentrations. ANOVA followed by Bonferroni’s posthoc test. * *p* < 0.05 vs. control. (**B**) Network analysis of the conditioned media proteome measured by flow cytometry-based assays, showing the fold change comparison of vascular inflammation molecules between untreated U1 and U937 conditioned media, where red shades indicate upregulation and blue shades indicate downregulation in U1 cells compared to U937 conditioned media. Squares indicate significant *p* values. (**C**–**H**) Protein levels in untreated U937 and U1 conditioned media in pg/ml, normalized to standard curve. Dots indicate biological replicate measurements. (**C**) PIGF, (**D**) TNFa, (**E**) SAA, (**F**) IGFBP-4, (**G**) MPO and (**H**) Cystatin C. N = 10/condition. Student’s *t* test *p* values are indicated.

These multi-cell systems were used to examine the impact of conditions that replicate the HIV-infected brain in the ART era, specifically how an environment created by latently infected myeloid cells, affect BBB. The latently infected environment was modeled by the incubation of BBB systems with conditioned media obtained from cultures of U1 promonocytes latently infected with HIV1, which have been derived from uninfected parent U937 promonocytes [74], used as controls. The characterization of the U1 conditioned media collected 48 h after cell split, and performed in 6 independent measures in duplicate, revealed a low baseline level of p24 with an average of 38.41 pg of p24/mL (±3.33). The latent state of the U1 cells was confirmed by incubation with latency reversal agents PEP005, iBET and phorbol myristate acetate (PMA), which significantly enhanced p24 levels (Figure 2A). The supernatants were further characterized using flow cytometry-based proteome assays, indicating differences in levels of a network of vasoactive and pro-inflammatory proteins between uninfected U937 and latent U1 supernatants (Figure 2B). For instance, U1 conditioned media contained increased levels of Placental Growth Factor (PIGF) (Figure 2C), tumor necrosis factor alpha (TNFα) (Figure 2D), insulin-like growth factor binding protein-4 (IGFBP4) (Figure 2F), myeloperoxidase (MPO) (Figure 2G), and Cystatin C (CST3) (Figure 2H). Serum Amyloid A (SAA) (Figure 2E) was also increased at a substantial level. On the other hand, inflammatory proteins such as the receptor for advanced glycation end products (RAGE), Osteopontin (SPP1) and CCL2 were significantly lower in U1 conditioned media compared to control U937 supernatants (*p* = 0.004, 0.05, 0.05, respectively) in agreement with the latency phenotype. The increase of proteins with described effects on the BBB suggests that the model is adequate to mimic the endothelial damage occurring in the brain environment in the context of latent HIV, and to test how cannabinoid signaling may interfere with those effects.

**Figure 3:**
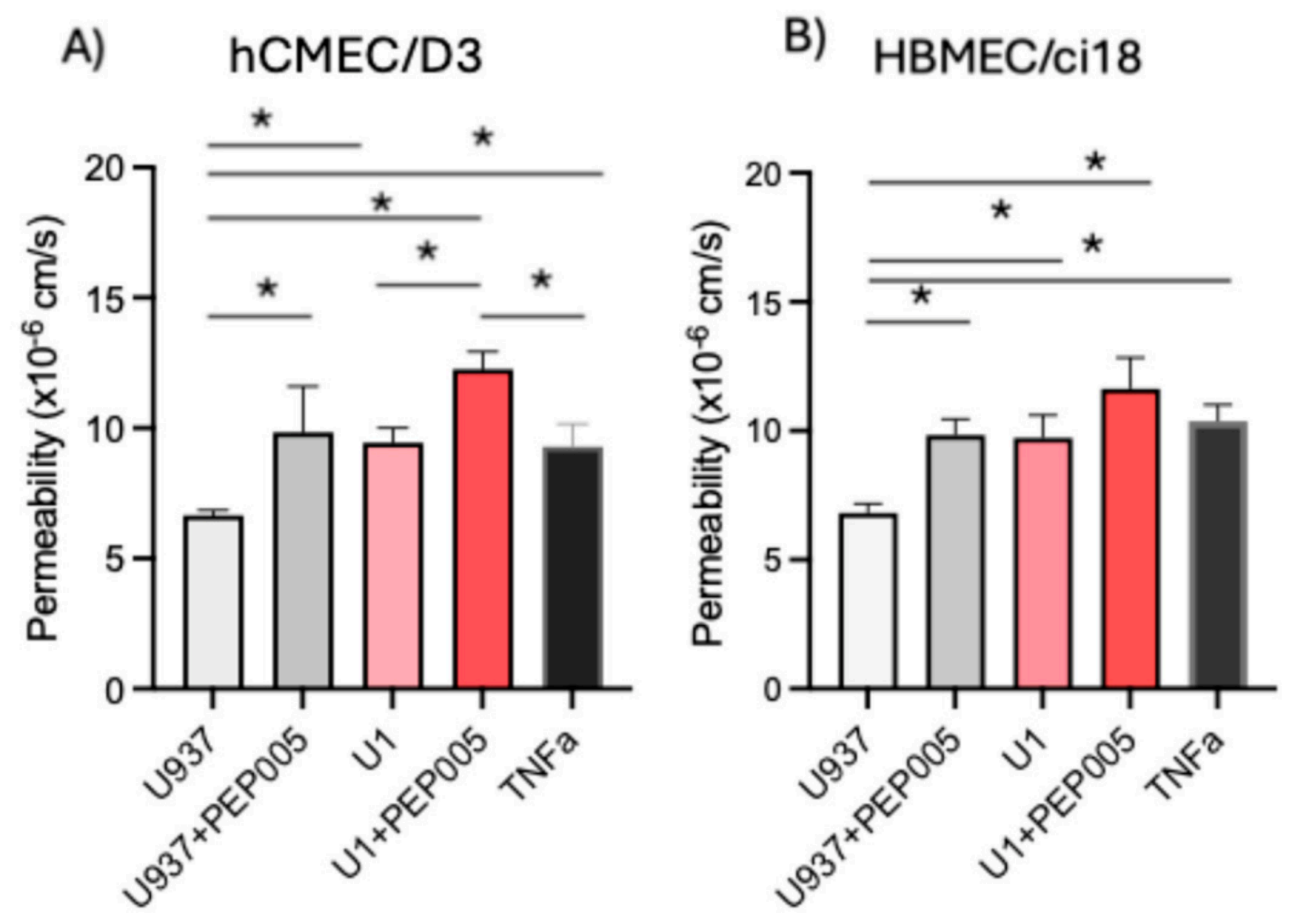
Effects of latent HIV conditioned media on BBB permeability. The endothelial cell lines (**A**) hCMEC/D3 and (**B**) HBMEC/ci18 were assembled with pericytes and astrocytes to test the effect of conditioned media from U937 (control) or U1 (latent) promonocytes. To control for the effect of virus in the conditioned media, U937 and U1 cells were treated with the latency reversal agent PEP005 (10 nM), or vehicle control. Recombinant TNFa 10 ng/mL was used as a positive control for the permeability to Cascade Blue-labeled 10 kDa dextran particles. Data are expressed as mean of values obtained from at least 3 independent experiments ± SEM. N = 3 biological replicates. * *p* < 0.05 on indicated comparisons. ANOVA followed by Bonferroni’s posthoc.

To further validate the model, we compared the effects of U1 conditioned media on the permeability to 10 kDa dextran molecules in multicellular BBB systems containing either one of the cerebrovascular endothelial cells. U937-conditioned media did not affect BBB permeability significantly in either cell system. However, in the presence of U1-conditioned media, significant disruption of the BBB integrity was observed in both hCMEC/D3 (Figure 3A) and HBMEC/ci18 (Figure 3B), as revealed by increased permeability to dextran (Figure 3) compared to U937-conditioned media. The BBB disruption by U1-conditioned media was similar to the damage triggered by TNFα, used as a positive control, and present in U1 supernatants (Figure 2). Importantly, conditioned media from U1 cells stimulated with the latency reversal agent PEP005 showed increased BBB permeability. In hCMEC/D3 cells U1 + PEP005 increased permeability significantly above the positive control TNFα, but not in HBMEC/ci18. However, in consideration to the >1000-fold increase in p24 caused by PEP005 (Figure 2A), the result suggests that the virus in the supernatant had a rather modest or no contribution to the increase in BBB permeability. Thus, the exposure to U1 conditioned media was deemed adequate as a model to mimic the BBB disruption in the context of latent HIV, such as in the CNS of PWH, and to test the effects of cannabinoids and their receptors in multicellular models containing the two cerebrovascular endothelial cell lines.

**Figure 4:**
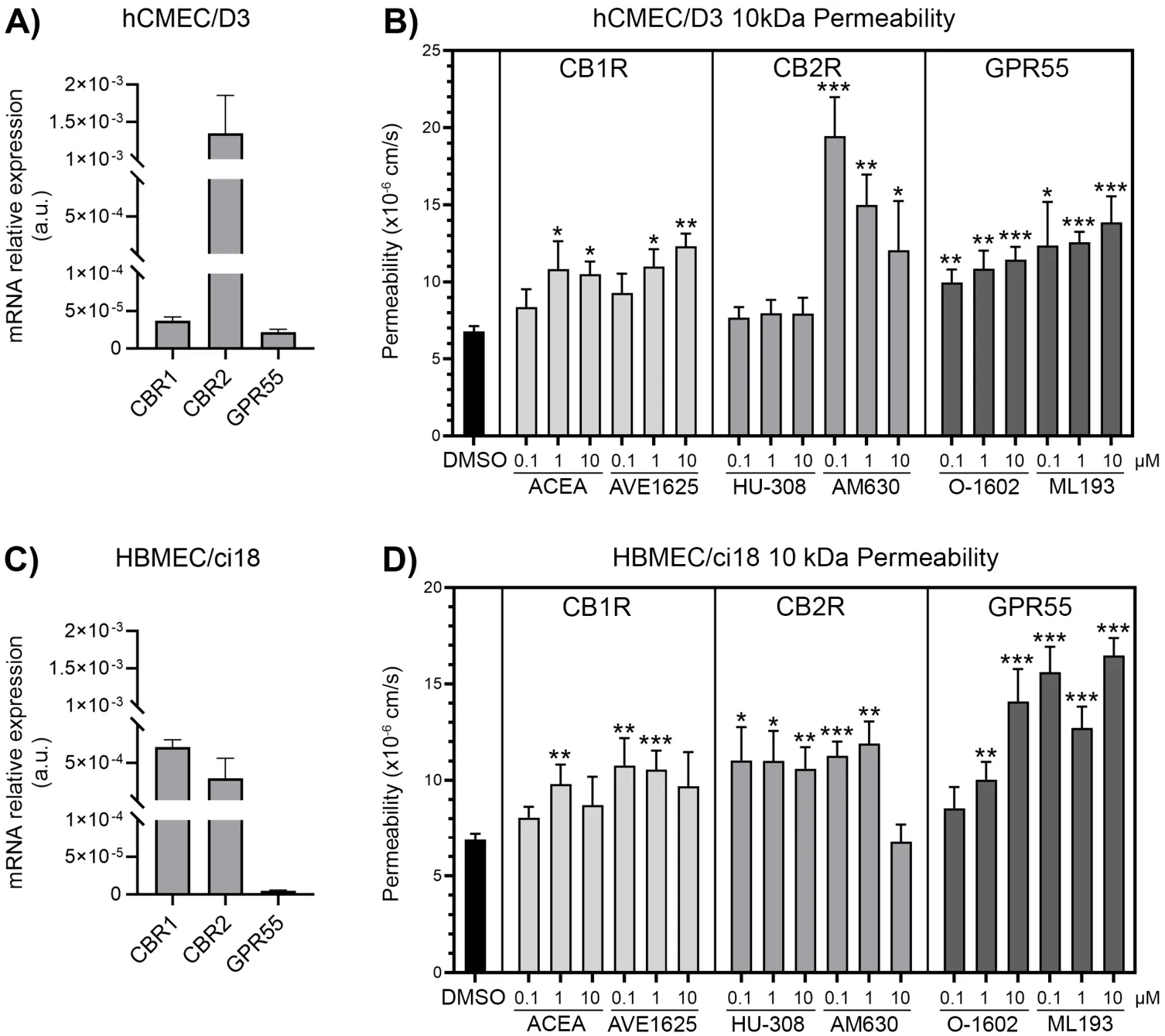
Endogenous cannabinoid receptor expression in hCMEC/D3 and HBMEC/ci18 cells and effects of cannabinoid receptor signaling on BBB permeability. (**A**) Relative mRNA expression of CB1R, CB2R and GPR55 in hCMEC/D3 normalized to GAPDH. (**B**) Effects of varying concentrations of agonists and antagonists of the indicated CBRs on BBB permeability to 10 kDa dextran in hCMEC/D3. (**C**) Relative mRNA expression of CB1R, CB2R and GPR55 in HBMEC/ci18 endothelial cells normalized to GAPDH. (**D**) Effects of varying concentrations of agonists and antagonists of the indicated CBRs on BBB permeability to 10 kDa dextran in HBMEC/ci18. DMSO was used as vehicle control for baseline effects. CB1R agonist ACEA and antagonist AVE1625, CB2R agonist HU-308 and antagonist AM630, and GPR55 agonist O-1602 and antagonist ML193 were provided at the indicated uM concentrations, for 24 h prior to the addition of dextran. Permeability was measured by fluorescence after 1 h. Data are expressed as mean of values obtained from 3 independent experiments ± SEM. N = 3 biological replicates. * *p* < 0.05, ** *p* < 0.01, *** *p* < 0.001 compared to DMSO controls, ANOVA followed by Bonferroni’s posthoc test.

First, we confirmed that the two endothelial cell lines express all the major cannabinoid receptors CB1R, CB2R and GPR55 (Figure 4A). Importantly, hCMEC/D3 cells had low transcriptional levels of CB1R, but high levels of CB2R. On the other hand, HBMEC/ci18 had higher CB1R expression but lower levels of CB2R compared to hCMEC/D3 cells. Both cell lines had very low expression of GPR55. We then screened the ability of cannabinoid receptor signaling to modify the BBB disruption outcomes in both BBB systems, regardless of the HIV latent environment. For this, we tested pharmacological agonists and antagonists to CB1R and CB2R, as well as GPR55 in both cell lines, and in control conditions (Figure 4B,C). The agonists and antagonists of CB1R and GPR55 all increased permeability of the BBB in both cell lines, suggesting that cannabinoid ligands have direct effects on endothelial cells. On the other hand, the agonist for CB2R HU-308 had no effect on hCMEC/D3 cells, while its antagonist drastically increased permeability (Figure 4B). However, this effect was not observed in HBMEC/ci18 cells in which permeability was increased by both CB2R agonist and antagonist (Figure 4C). This result suggests that potential benefits to the BBB may result from CB2R signaling, facilitated by the agonist HU-308 in hCMEC/D3, but not in HBMEC/ci18, and that the molecular difference in CB2R signaling between these two endothelial cell lines may provide a potential mechanism for understanding different effects observed in human vascular integrity markers in response to cannabis use, and in the context of HIV. The lack of effect of HU-308 alone was contrasted with the increase in permeability caused by AM630. Interestingly, incubation with CB1R agonist ACEA as well as the antagonist AVE1625, and with the GPR55 agonist O-1602 and antagonist ML-193 caused an increase in vascular permeability in both cell lines, suggesting that signaling through cannabinoid receptors other than CB2R on endothelial cells may enhance vascular injury (Figure 4B,C).

**Figure 5:**
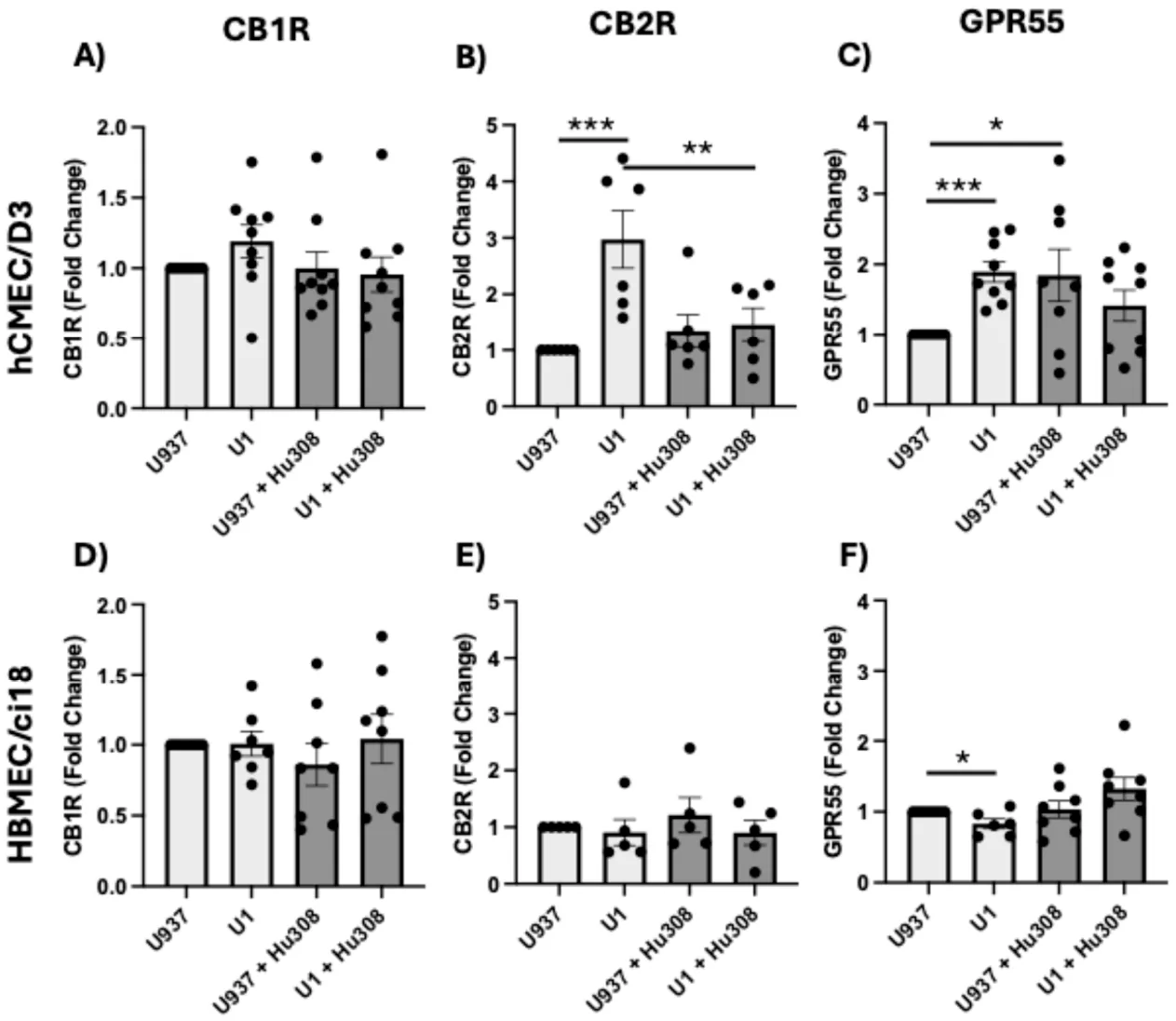
Latent HIV-infected environment and CB2R activation differentially affect cannabinoid receptor expression in hCMEC/D3 and HBMEC/ci18 cells. Fold change from mRNA baseline expression levels of (**A**,**D**) CB1R, (**B**,**E**) CB2R and (**C**,**F**) GPR55 in (**A**–**C**) hCMEC/D3 and (**D**–**F**) HBMEC/ci18 cells after 24 h pre-incubation with DMSO or HU-308 (10 μM) along with exposure to untreated U937 or U1-conditioned medium. Data are expressed as mean of values obtained from 3 independent experiments ± SEM. N = 10–12/condition. * *p* < 0.05, ** *p* < 0.01, *** *p* < 0.001 for indicated comparisons. ANOVA, Bonferroni’s posthoc.

We used the validated U1 conditioned media model to mimic HIV latent conditions to test whether the HIV latent environment characterized above modifies the levels of cannabinoid receptors on the endothelial cell lines and whether the CB2R agonist HU-308 modifies these effects (Figure 5). In hCMEC/D3 cells, U1 conditioned media did not affect the expression of CB1R in the presence or absence of HU-308 but significantly increased the expression of CB2R and GPR55. The pretreatment with HU-308 partially prevented the change in levels of CB2R caused by U1-conditioned media. In addition, while HU-308 alone increased GPR55, the addition of U1 conditioned media to cells pretreated with HU-308 maintained GPR55 transcription at control levels (Figure 5A). In HBMEC/ci18, neither U1 conditioned media nor HU-308 affected CB1R or CB2R expression. U1 conditioned media caused a modest but significant decrease of GPR55 transcription, which was prevented by the pretreatment with HU-308 (Figure 5B). These data suggest that cerebral endothelial cells can differ in their expression of cannabinoid receptors, and that the environment containing HIV-latent myeloid cells modulates the levels of cannabinoid receptors differentially in the two cell lines. It also indicates that the HIV latent environment causes detrimental effects to endothelial health, and that signaling via CB2R can prevent these damaging effects in correlation with its transcriptional upregulation.

**Figure 6:**
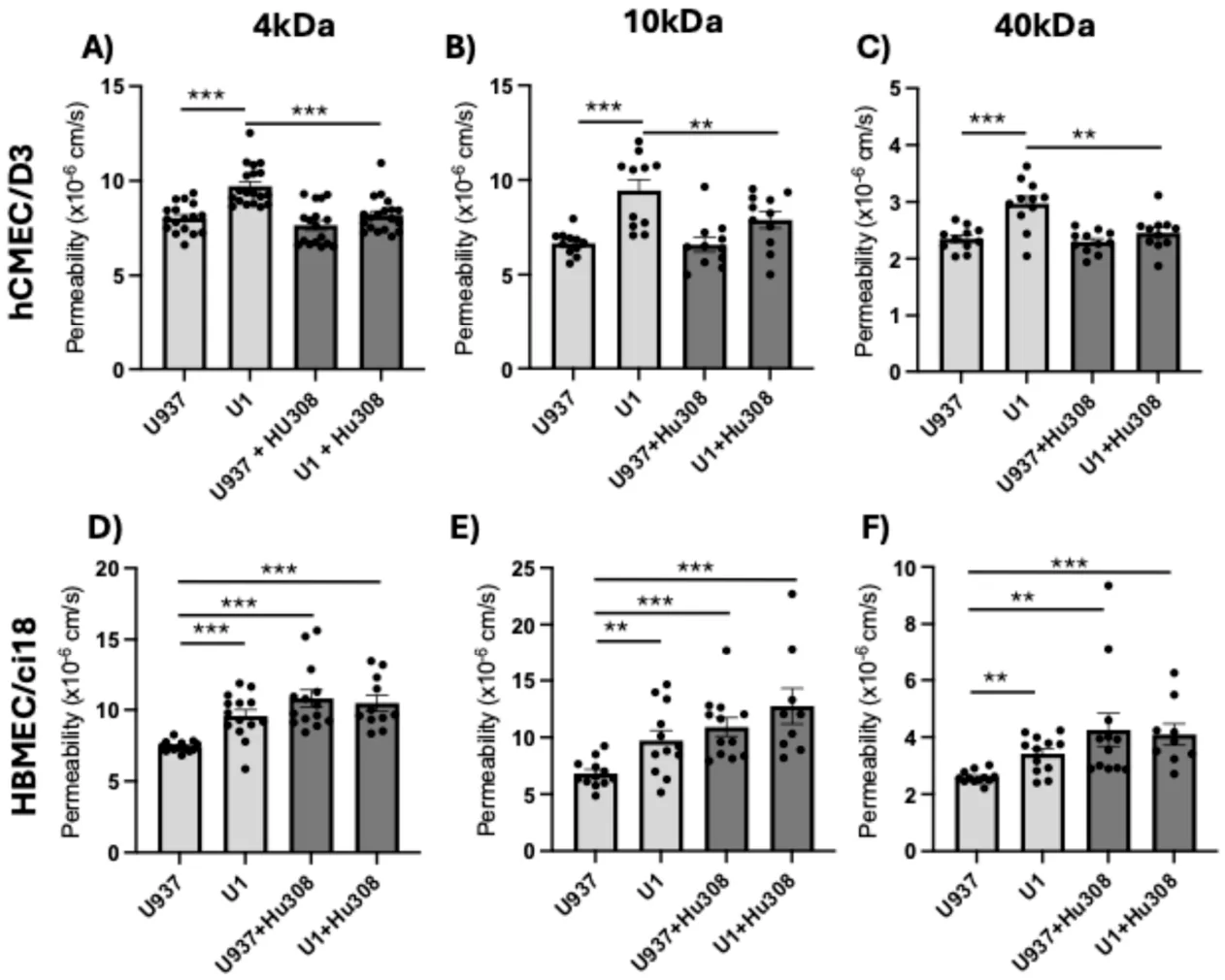
Effect of CB2R agonist HU-308 on hCMEC/D3- and HBMEC/ci18-containing BBB permeability. Barrier permeability to 4 (**A**,**D**), 10 (**B**,**E**) and 40 kDa (**C**,**F**) in multicellular BBB cultures containing hCMEC/D3 (**A**–**C**) or HBMEC/ci18 (**D**–**F**) endothelial cells, 24 h following the addition of untreated control U937 conditioned medium or U1 latent HIV-conditioned medium, in the presence of DMSO or 10 μM HU-308. Data are expressed as mean of values obtained from 3 independent experiments ± SEM. N = 10–12/condition. ** *p* < 0.01, *** *p* < 0.001 for indicated comparisons, ANOVA followed by Bonferroni’s posthoc test.

**Figure 7:**
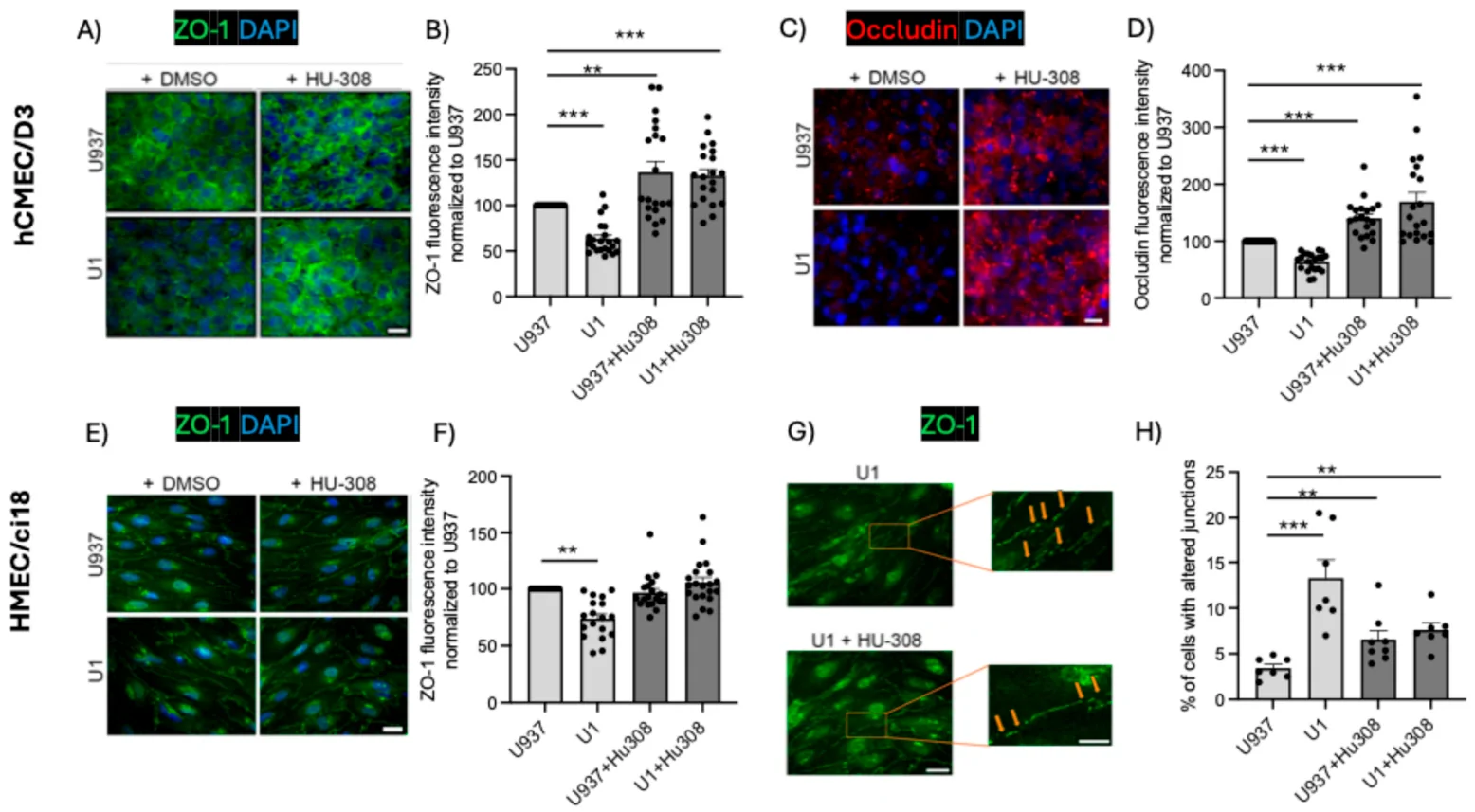
Effect of CB2R agonist HU-308 on the tight junction proteins and integrity in BBB systems containing hCMEC/D3 or HBMEC/ci18. Tight junction proteins were measured in polyculture BBB systems containing (**A**–**D**) hCMEC/D3 or (**E**–**H**) HBMEC/ci18 endothelial cells, 24 h in the presence of control U937 or latent HIV U1-conditioned media, and 0.1% DMSO or 10 μM HU-308. (**A**) Representative images of ZO-1 (green) and Dapi nuclear staining (blue) on hCMEC/D3 cells. Scale bar = 10 μm. (**B**) Quantification of ZO-1 fluorescence intensity by ImageJ/Fiji (NIH), normalized by area and to control conditioned media. Data are the mean of 3 independent experiments in triplicate ± SEM. ** *p* < 0.01, *** *p* < 0.001 compared to U937 untreated controls. (**C**) Representative images of Occludin (red) and Dapi (blue) on hCMEC/D3 cells in BBB polycultures. (**D**) Quantification of Occludin fluorescence intensity measured in ImageJ/Fiji (NIH), normalized by area and to untreated control conditioned media. (**E**) ZO-1 expression (green) on HBMEC/ci18 polycultures exposed to untreated control U937-conditioned medium or U1 latent HIV-conditioned medium, and 0.1% DMSO or 10 μM HU-308. Scale bar = 10 μm. (**F**) Quantification of ZO-1 fluorescence intensity normalized by area and to U937 control conditioned medium. Data are the mean of 3 experiments in triplicate ± SEM, for a total N = 12–18/condition. The total number of quantified cells was 293 in U937, 286 in U1, 407 in U937 + HU-308 and 331 in U1 + HU307). ** *p* < 0.01, *** *p* < 0.001 compared to U937 untreated controls. (**G**) Magnification of HBMEC/ci18 image with untreated U1-conditioned media, allowing the observation of gaps in tight junction protein expression (orange arrows). Scale bar, 5 µm. (**H**) Number of cells showing membrane gaps on ZO-1 distribution by condition. ** *p* < 0.01, *** *p* < 0.001 for indicated comparisons, ANOVA followed by Bonferroni’s posthoc test.

We next probed the effects of CB2R activation with HU-308 on the increase in barrier permeability caused by the latent HIV-conditioned medium. In hCMEC/D3-containing cultures, the stimulation of CB2R with HU-308 protected against the increase in permeability caused by U1 conditioned media. The protective effect was observed with all sizes of dextran particles (Figure 6A). Consistent with findings in Figure 3, immunohistochemistry analysis demonstrated that U1 conditioned media leads to a reduction in expression of tight junction proteins ZO-1 and occludin (Figure 7). However, hCMEC/D3-containing cultures pretreated with HU-308 had a higher basal level of these proteins and were protected from the decrease caused by U1 conditioned media (Figure 7).

In contrast, in HBMEC/ci18-containing BBB systems the addition of HU-308 alone or together with U1-conditioned media worsened BBB permeability (Figure 6D–F). Curiously, despite increasing permeability in HBMEC/ci18 (Figure 6B), HU-308 prevented the loss of ZO-1 in those cells (Figure 7E,F). However, the inspection of ZO-1 distribution patterns on the surface of HBMEC/ci18 cells indicated that HU-308-mediated recovery of ZO-1 levels only partially decreased gaps in tight junctions caused by U1-conditioned media (Figure 7G,H). This was confirmed by the quantification of the relative number of cells with membrane gaps (Figure 7H).

**Scheme 1:**
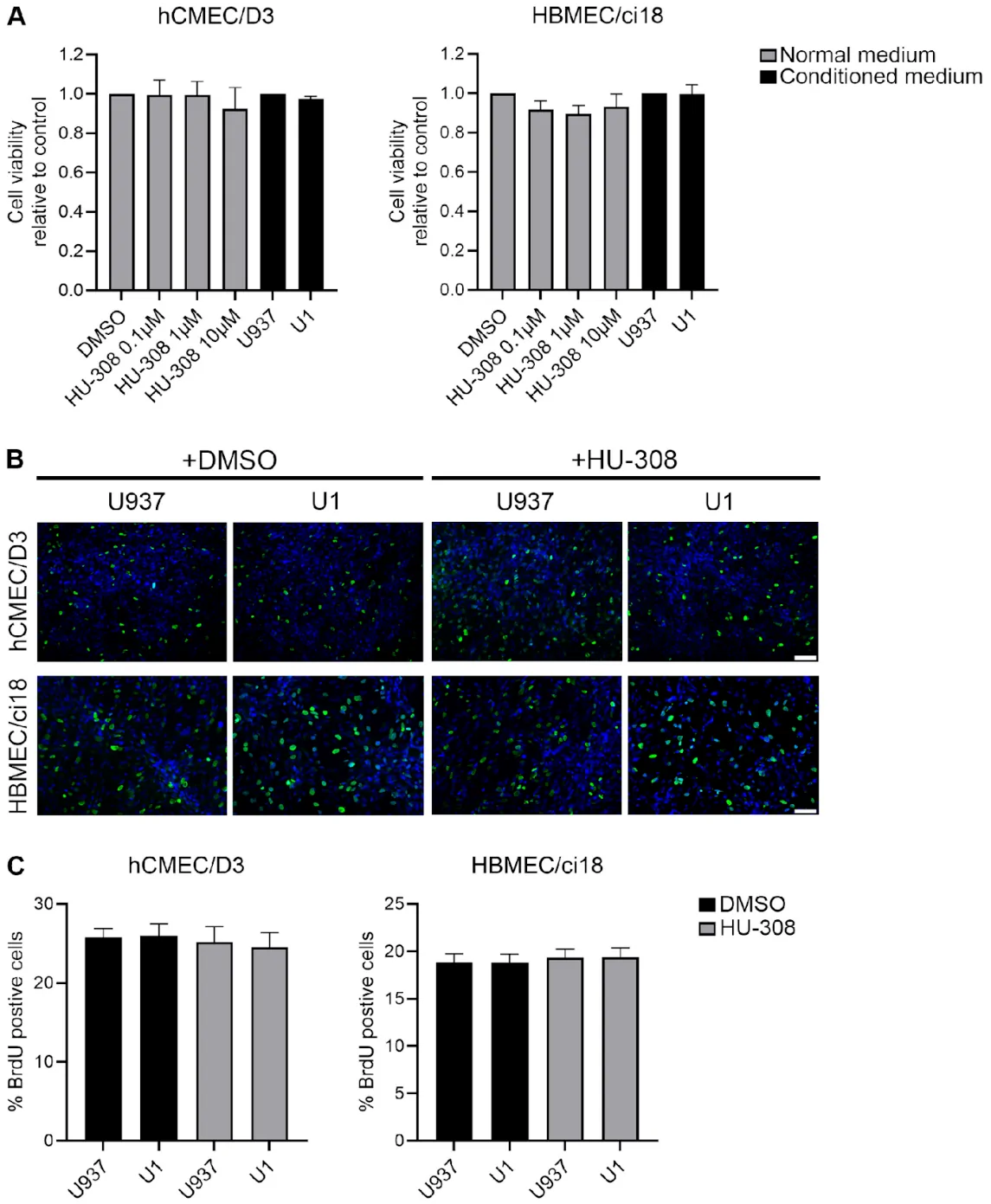
Cellular viability and proliferation of hCMEC/D3 and HBMEC/ci18 cells following exposure to promonocytes conditioned media and HU-308. (**A**) Cell viability in hCMEC/D3 and HBMEC/ci18 by XTT following increasing concentrations of HU-308, with untreated control U937- or U1- latent HIV-conditioned media. (**B**) Representative images of BrdU incorporation (green) and Dapi nuclear staining (blue) in hCMEC/D3 and HBMEC/ci18 monolayers with conditioned media in presence of 0.1% DMSO or 10 μM HU-308 for 24 h. Scale bar = 50 μm. (**C**) Percentage of proliferating hCMEC/D3 and HBMEC/ci18 after exposure to 10 μM HU-308, measured by BrdU incorporation. Data are expressed as mean of 3 independent experiments ± SEM, for a total N = 10–12/condition. No statistical significance was identified. ANOVA followed by Bonferroni’s posthoc test.

Importantly, the effects leading to hCMEC/D3 recovery and HBMEC/ci18 damage by HU-308, particularly in the context of HIV-latent conditioned media, were not due to changes in the proliferation and survival of these cells (Scheme 1).

**Figure 8:**
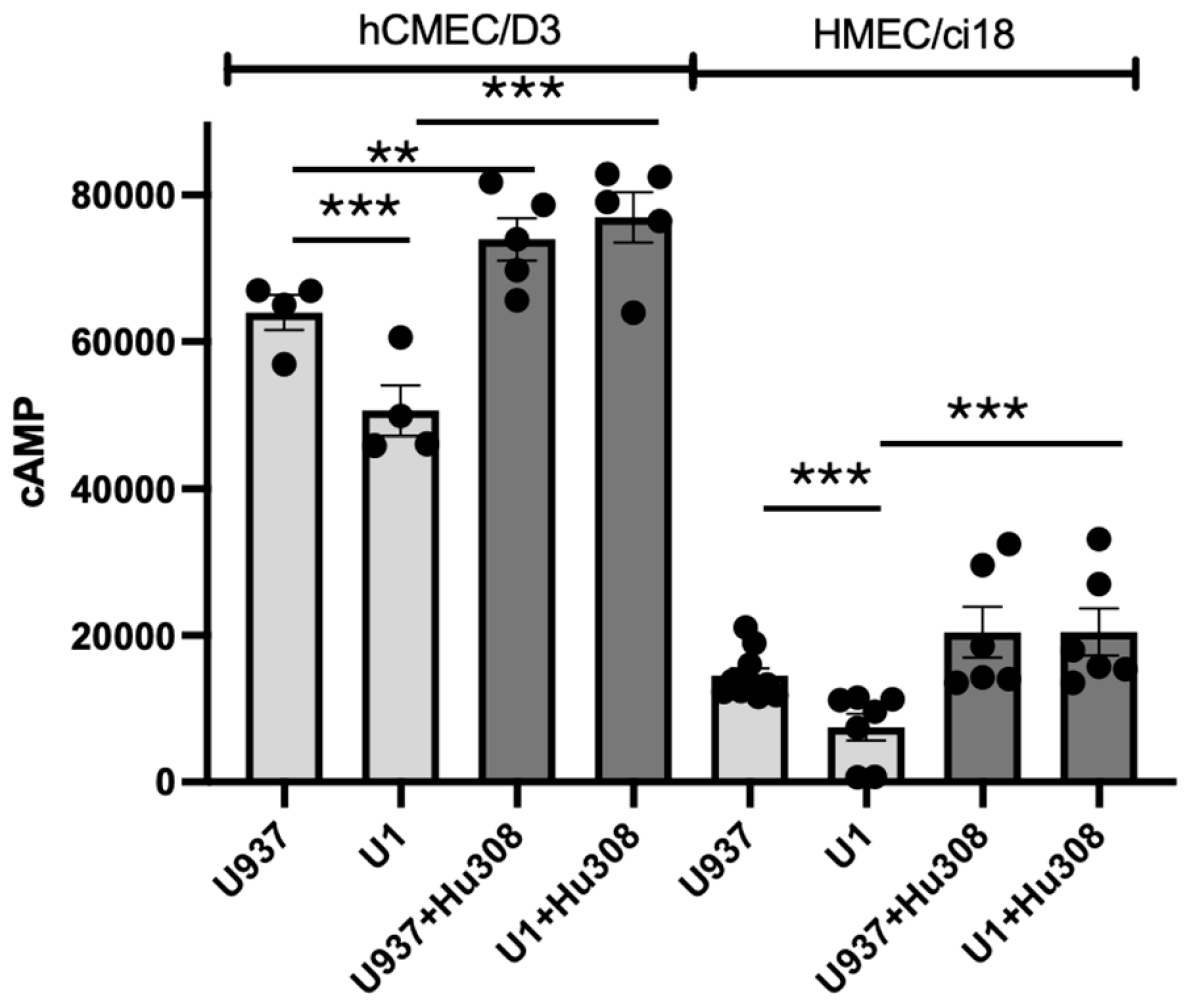
Levels of forskolin-stimulated cyclic AMP in hCMEC/D3 or HBMEC/ci18 cells following untreated U937 or U1-conditioned media, and effects of CB2R activation. The endothelial cultures were incubated with U937- or U1-conditioned media, in the presence of DMSO or 10 μM of HU-308. The cells were processed for the detection of cAMP measured by fluorescence and calculated with a standard curve. Data are expressed as mean of 3 independent experiments ± SEM, with represented individual replicates for a total N = 5–10/condition. ** *p* < 0.01, *** *p* < 0.001 compared to U937 untreated controls. ANOVA followed by Bonferroni’s posthoc test.

To begin unraveling the mechanisms by which HU-308-induced CB2R signaling prevents hCMEC/D3 but not HBMEC/ci18 endothelial barrier disruption we evaluated whether there are defects in their G-PCR response capacity using forskolin-induced cAMP upon conditions of HIV latency and CB2R activation in these cells (Figure 8). Interestingly, hCMEC/D3 produced 50–200-fold more cAMP in response to forskolin stimulation compared to HBMEC/ci18 in untreated conditions (Figure 8). In addition, hCMEC/D3, but not HBMEC/ci18, showed significantly increased sensitivity of cAMP response to forskolin in the presence of HU-308 alone. U1-conditioned media promoted forskolin desensitization in both cell lines, and HU-308 promoted recovery. This suggests that despite the drastically lower achievable forskolin-induced cAMP levels in HBMEC/ci18 and overall lower expression of CB2R, the CB2R agonist is able to trigger a response in these cells, suggesting that the HIV-latent environment does not induce a defective response.

This difference in baseline cAMP response to forskolin is aligned with differences in the transcriptome profile within GPCR pathway-associated genes.

**Figure 9:**
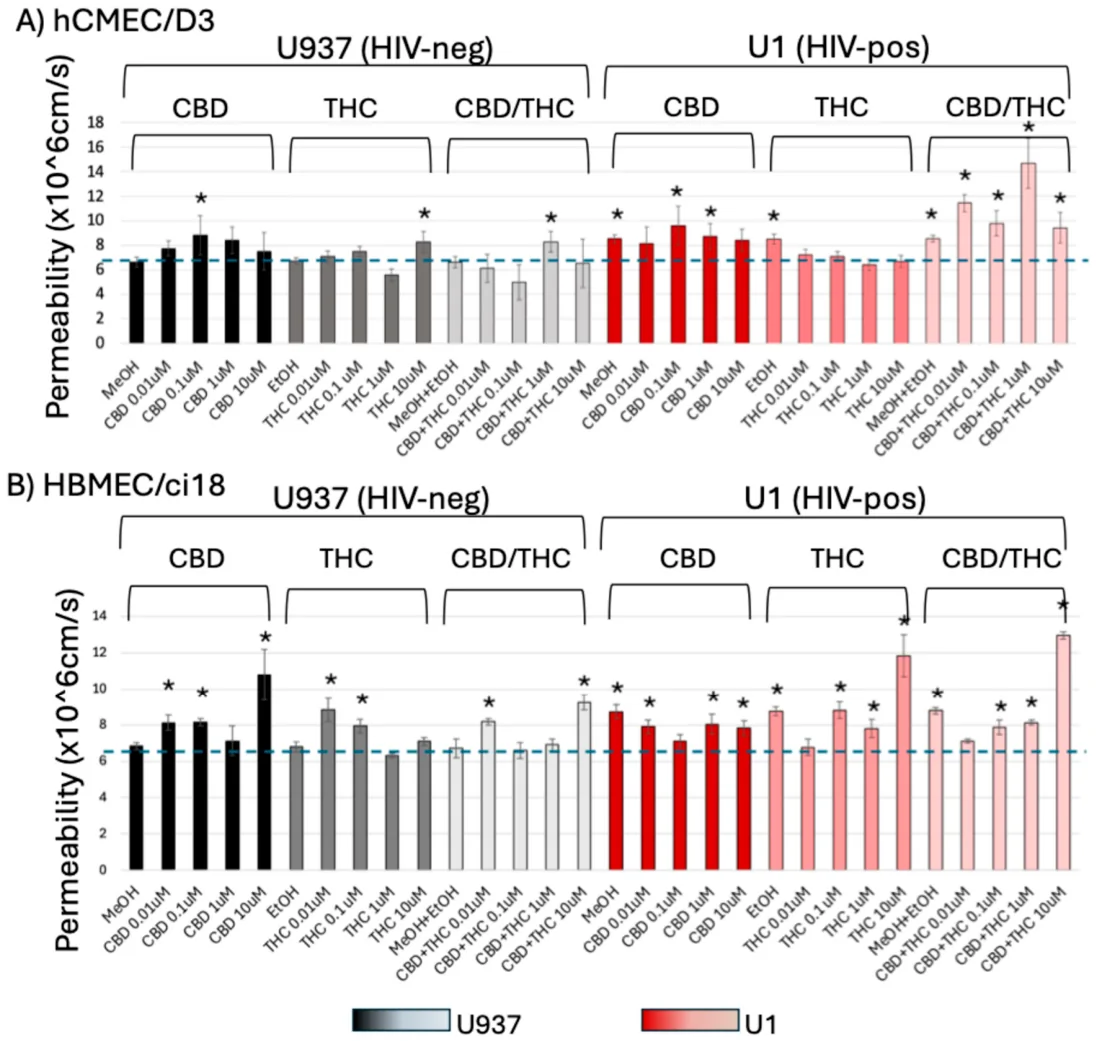
Effects of plant-derived cannabinoids on BBB systems’ permeability of hCMEC/D3 or HBMEC/ci18 cells exposed to untreated U937 or U1-conditioned media. (**A**,**B**) Permeability to 10kDa dextran in multicellular BBB systems containing hCMEC/D3 cells (**A**) and HBMEC/ci18 cells (**B**). Data are expressed as the mean of 3 independent experiments in triplicate ± SEM. * *p* < 0.05, compared to U937 untreated controls, ANOVA followed by Bonferroni’s posthoc test.

To further confirm the distinct CB2R response patterns between the two cell lines, we used complete BBB systems and plant-derived cannabinoids THC and/or CBD. The cultures were tested for changes in the BBB integrity by measuring permeability to 10kDa dextran (Figure 9A,B). Consistent with the experiments using the selective CB2R agonist HU-308, HBMEC/ci18 cells had more disruption of BBB integrity than hCMEC/D3 cells with both CBD or THC alone (Figure 9A,B). Moreover, HBMEC/ci18 cells were not protected from the damaging effects of U1 conditioned media by THC or CBD (Figure 9B), while in hCMEC/D3 cells pretreatment with THC prevented the increase in permeability that occurred after exposure to U1-conditioned media (Figure 9A). However, CBD alone did not protect hCMEC/D3 cells against U1-latent media and even aggravated the increase in permeability when combined with THC (Figure 9A).

**Figure 10:**
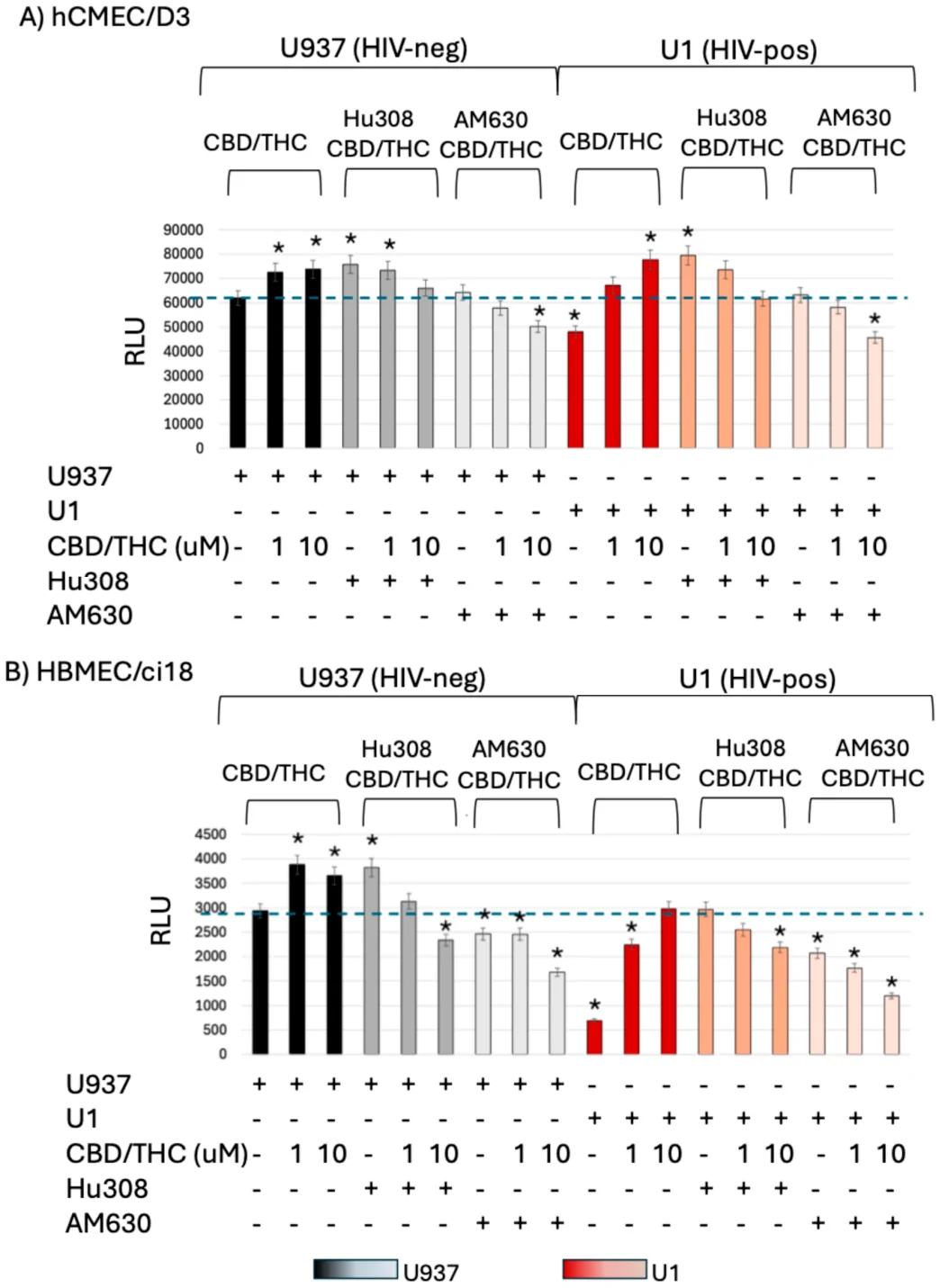
Effects of plant-derived cannabinoids on forskolin-sensitive cyclic AMP induction in hCMEC/D3 or HBMEC/ci18 cells exposed to untreated U937 or U1-conditioned media. Cells were treated with CBD/THC alone or together with Hu-308 or AM630. (**A**,**B**) Normalized cAMP levels in single cultures of hCMEC/D3 (**A**) or HBMEC/ci18 (**B**) endothelial cells. Data are expressed as the mean of 3 independent experiments in triplicate ± SEM. * *p* < 0.05 compared to U937 untreated controls, ANOVA followed by Bonferroni’s posthoc test.

Also, in agreement with the previous experiments, CBD and THC increased forskolin-induced cAMP in both cell lines, in a CB2R-dependent manner, as indicated by the effects of the CB2R antagonist AM630, particularly when cannabinoids were added at higher doses (Figure 10A,B). Yet, the levels of cAMP induced by the combined cannabinoids were up to 80-fold higher in hCMEC/D3 cells (Figure 10A) compared to HBMEC/ci18 (Figure 10B). Interestingly, the combination of HU-308 and THC/CBD in the context of U1-conditioned media decreased cAMP in a dose-dependent manner (Figure 10A,B) in correlation with increased permeability in both cell lines (Figure 9). This raises the possibility of a potential reversal of the benefits via CB2R in pro-inflammatory conditions where other cannabinoid receptors are also activated. While cAMP is enhanced via selective CB2R activation, this result suggests that a signaling threshold is needed for beneficial effects and that the benefits may be overridden by the binding of cannabinoids to other receptors, masking or reversing benefits in the context of inflammation.

**Figure 11:**
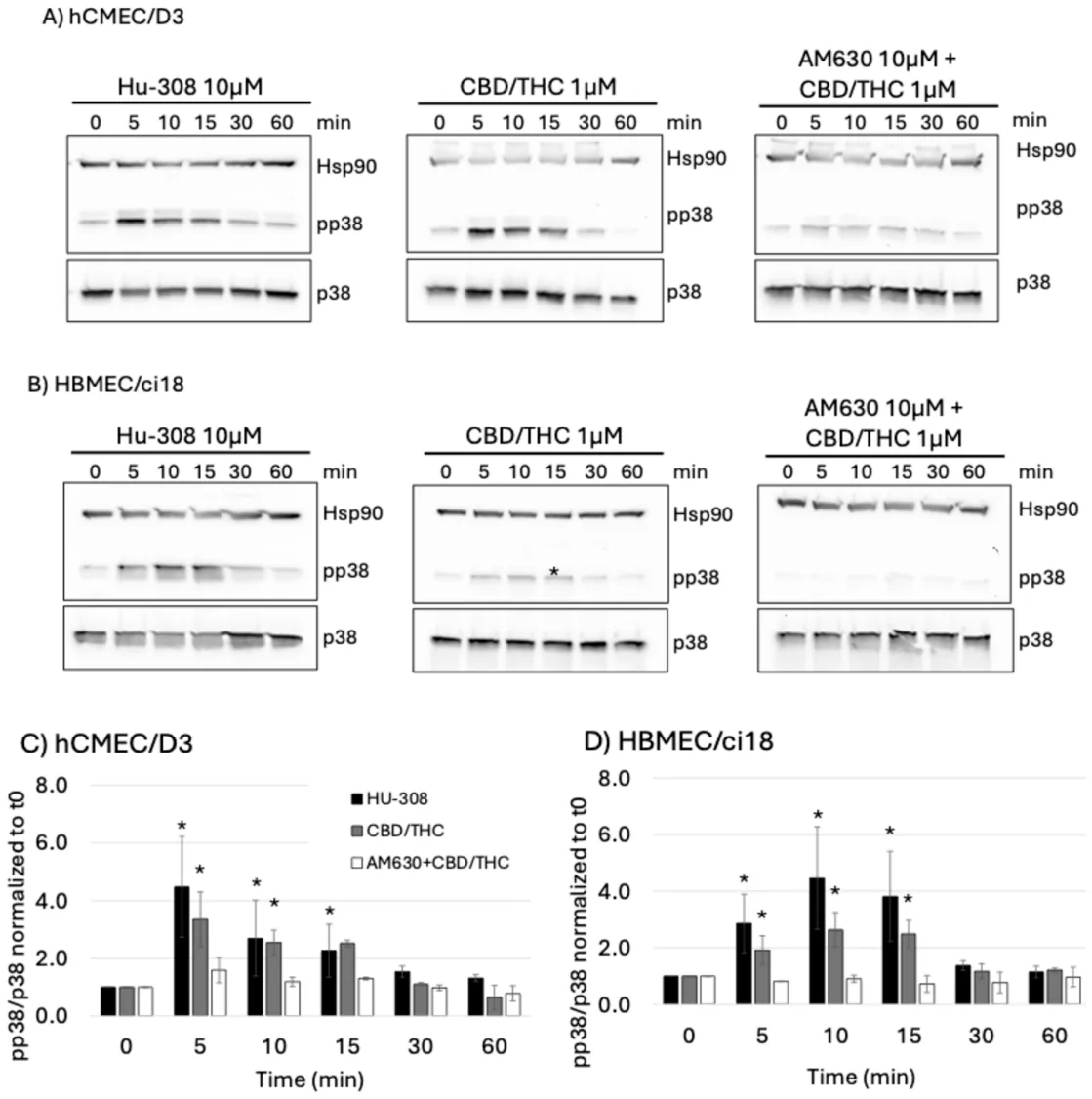
HU-308 and CBD/THC induce rapid, transient p38 MAPK phosphorylation in brain endothelial cells, blocked by CB2 antagonism. (**A**,**B**) Representative immunoblots of phosphorylated p38 (pp38), total p38 and Hsp90 (loading control) in hCMEC/D3 (**A**) and HBMEC/ci18 (**B**) treated with HU-308 (10 µM), a CBD/THC combination (1 µM), or CBD/THC (1 µM) following pre-treatment with the CB2R reverse agonist/antagonist AM630 (10 µM), over 0–60 min. (**C**,**D**) Densitometric quantification of pp38 normalized to total p38 and expressed relative to the untreated (time = 0 min) control for hCMEC/D3 (**C**) and HBMEC/ci18 (**D**); bars show mean ± SD of n = 3 independent experiments. * *p* < 0.05 versus the 0 min control (ANOVA followed by Bonferroni’s test).

To investigate whether CBD/THC activates p38 MAPK signaling in cerebrovascular endothelial cells and whether this response is CB2R-mediated, we treated hCMEC/D3 and HBMEC/ci18 cells with CBD/THC (1 µM each) over a 60-min time course and assessed phospho-p38 (pp38) levels by western blot, normalized to the loading control Hsp90 and to total P38 (Figure 11). In hCMEC/D3 cells, both HU-308 and CBD/THC induced a rapid and robust increase in pp38, peaking at 5 and 10 min compared to the t = 0 baseline, followed by a progressive decline to near-baseline levels by 30–60 min (Figure 11A,C). This transient activation profile is consistent with a rapid, self-limiting p38 MAPK response downstream of cannabinoid receptor engagement. Despite a similar response to HU-308, HBMEC/ci18 cells exhibited a more attenuated CBD/THC response that reached a modest peak at 5 min and declined steadily thereafter, approaching negligible levels by 60 min, suggesting a detectable effect, but a lower capacity for sustained pp38 MAPK activation in this cell line (Figure 11B,D). To assess the contribution of CB2R to these responses, cells were pre-incubated with AM630 (10 µM), a CB2R inverse agonist/antagonist, prior to CBD/THC exposure. In hCMEC/D3 cells, AM630 co-incubation substantially attenuated the pp38 response, reducing peak values about five-fold lower than CBD/THC alone, with a similar gradual decline to baseline by 60 min (Figure 11A,C). In HBMEC/ci18 cells, AM630 co-incubation effectively abolished the pp38 signal across all timepoints, with ratios remaining near zero throughout the time course (Figure 11B,D), indicating that the residual p38 MAPK activation observed with CBD/THC alone in this line is largely CB2R-associated. The weaker response to CBD/THC in HBMEC/ci18 compared to hCMEC/D3 may suggest that other cannabinoid receptors may cross regulate the CB2R response in those cells, indicating that CB2R is involved, but may be not sufficient to elicit a response aligned with benefit in that cell line.

Taken together, these data demonstrate that CBD/THC activates p38 MAPK in both cerebrovascular endothelial cell lines, but with different kinetics and magnitude. The response is stronger and more sustained in hCMEC/D3 than in HBMEC/ci18, and is CB2R-associated in both lines, as evidenced by its attenuation or abolition following AM630 pre-treatment. These findings suggest that the differential CB2R signaling capacity previously observed between these cell lines extends to MAPK pathway activation, with potential implications for endothelial inflammatory and barrier-regulatory responses to cannabinoids.

## 4. Discussion

With the use of conditioned media from HIV-latently infected promonocytes to mimic the HIV- infected brain environment in the ART era, we demonstrated that despite low levels of virus and low levels of pro-inflammatory and vasoactive components, such media causes a significant disruption of integrity in two human BBB cell line systems, hCMEC/D3 cells [75,76] and HBMEC/ci18 [77,78]. Conditioned media from the uninfected parent promonocytes (U937) did not affect BBB integrity, validating our in vitro system as an appropriate model for studying BBB disruption in the context of HIV.

The loss of vascular integrity in both cell lines was associated with decreased expression of TJ proteins such as ZO-1, and a significant increase in the permeability to dextran particles. Interestingly, in the context of an HIV environment, these two cell lines demonstrated opposing responses to the CB2R agonist HU-308. In hCMEC/D3, HU-308 prevented ZO-1 loss and decreased permeability to large molecules, suggesting the ability to protect and promote BBB integrity. By contrast, in HBMEC/ci18 cells, HU-308 recovered ZO-1 expression to baseline levels but further increased permeability in all conditions due to an inability to redistribute tight junctions correctly. The beneficial versus damaging HU-308 effects between the two cell lines aligned with significant differences in the levels of CB2R and the ability to build a sufficiently high cAMP level in response to forskolin stimulation. The decrease in cAMP caused by the HIV-environment was however corrected to baseline by the CB2R selective agonist in both cell types, and prevented by the selective antagonist, suggesting that the receptor is responsive in both cell lines, and that cAMP production is triggered via CB2R activation.

Whether residual viral particles in the latent cells-conditioned media can cause BBB disruption is an interesting question. It important that we specifically chose conditioned media from HIV-latently infected promonocytes (rather than replication-competent virus) to model the low-level, ART-era brain environment in which chronic BBB disruption and neuroinflammation persist despite viral suppression. Even upon the criticism that U1 cells do not transcribe a productive virus, HIV peptides such as Tat can perturb endothelial health [79]. However, the drastic increase in p24 by latency reversal agents, such as PEP005, indicate that latent state viral transcription does not translate to proportional damage. Besides, the incubation of uninfected U937 promonocytes with the same latency reversal resulted in a similar increase in BBB permeability, suggesting that the activation state of the target cells is critical to generate vasoactive effects regardless of the infection. Several prior studies have shown that cerebrovascular endothelial cells are strong gatekeepers and regulators of HIV infection, not likely permissive to infection, yet they are affected by HIV peptides such as Tat, Nef and gp120 and by chronic inflammation [79,80,81]. We thus hypothesize that the damage to the BBB in our model is indirect, in part by residual viral peptides, or due to vasoactive cytokines present in the supernatant. More studies are needed to clarify the contribution of components in the supernatant.

The protective effects of CB2R expression and its downstream signaling, specifically in endothelial cells where it appears to promote vascular health, suggests high clinical importance. Our findings provide a motivation to identify individuals who will benefit from cannabinoids and suggest that it might be beneficial to explain cannabis effect differences among PWH [56]. It is important to acknowledge studies that have linked chronic heavy use, particularly smoked cannabis, to increased CVD risk [42], possibly confounded by the mode of administration rather than cannabinoid receptor pharmacology per se. The receptor-selective agonist approach we use (HU-308 as a CB2R-selective compound) is intended to disentangle receptor-specific effects from the complex pharmacology of whole cannabis, and to guide its specific therapeutic potential.

The control of endothelial permeability by cAMP levels has been previously reported, where higher levels are able to increase tight junction proteins [82,83,84]. Moreover, cAMP has been regarded as an endothelial barrier stabilizer in inflammatory conditions [82,85]. The highest achievable cAMP levels in the two endothelial cell lines may explain different protective responses in the context of a latent HIV environment. One potential mechanism has been explored in hCMEC/D3 cells, where stimulation of adenosine receptors induces a Ca^2+^ influx by opening cyclic nucleotide-gated channels in a cAMP-dependent manner, causing formation and coupling of new gap junction plaques [86].

It is important to note that disturbing other cannabinoid receptors such as CB1R and GPR55 may lead to an increase in vascular permeability in both cell lines. The results of increased permeability in hCMEC/D3 cells stimulated with HU-308, THC and CBD simultaneously in the context of HIV latent supernatants indicates an interplay between these receptors, where excessive ligand availability and the binding to additional receptors may override the benefits of CB2R signaling, potentially due to a priming effect revealed by increased sensitivity to forskolin [87]. A decrease in net activation because of competitive binding of agonists or antagonists has been described in other GPCRs, either due to blocking or due to changes in receptor state or conformation [88,89].

One possible explanation is that CB2R signaling is largely saturated at baseline in hCMEC/D3, consistent with high constitutive receptor tone and/or receptor reserve. AM630 is documented to act as an inverse agonist at CB2R rather than a purely neutral antagonist in some systems [90], actively suppressing constitutive signaling; if hCMEC/D3 barrier integrity depends on such ligand-independent CB2R tone, then AM630 would unmask that dependency by shutting off signaling unrelated to the agonist and producing the observed drastic permeability increase, disproportionate relative to the lack of effect of HU-308 alone. Distinguishing constitutive tone from inverse agonism directly, for example using a neutral CB2R antagonist or a receptor knockdown remains an important follow-up.

To date, more than 150 minor cannabinoids comprising less than 1% of the total dry mass of the plant, have been identified [91,92,93], indicating a complex pharmacology and potentially further increasing the range of responses in translational and real-world settings, where variability may also depend on cannabis processing and formulation, and the global diversity of HIV subtypes. Yet, the differential responses to CB2R and its activation dynamic in cerebrovascular endothelial cells may explain previously observed differences in plasma levels of vascular inflammatory markers in humans in the context of HIV compared to uninfected cannabis users [56], and depending on cannabis use patterns [94], which are predictive of neurocognitive outcomes. Our in vitro results performed in cerebrovascular endothelial cells suggest that levels of cAMP and activation thresholds may predict whether CB2R signaling will elicit beneficial outcomes culminating in recovery of the BBB integrity. The results also suggest that CB2R selective agonists may prove a more reliable way to deliver improvements in BBB function due to specificity. Another CB2R agonist, PM289, has been shown to improve barrier leakiness in a model using TNFα-induced damage in hCMEC/D3 cells in correlation with a moderation of the increase in NFkB availability [66].

This study has limitations due to the use of immortalized cell lines, which were favored here to minimize variability complicating the analysis of mechanisms, but limit translational conclusions beyond speculation. Studies on primary cerebrovascular endothelial cells from different sources must be performed to build upon these observations with data on populational diversity including genotype and cannabis use patterns. Yet, the value of the experimental models relies on the ability to dissect molecular pathways. The data showing distinct responses in two cerebrovascular endothelial cell lines also clearly warrants caution when making translational extrapolations from cell line experiments. Another limitation is related to the emulation of the brain environment using conditioned media from latently infected cells. However, the rigorous proteome and viral characterization with the production of endothelial damage constituted a controlled model to screen the molecular effects of cannabinoid compounds incorporating a diversity of models. The use of a well-characterized conditioned media was favored to eliminate the effects of cannabinoids on infected mostly myeloid target cells in the system and allow isolation and control of BBB responses via targeted receptors, while serving as a surrogate for the full glial-endothelial crosstalk occurring in vivo. Studies using similarly well-characterized human-derived plasma and CSF are needed. In addition, our findings were obtained using a latent HIV-1 (subtype B) promonocyte model. The effects of HIV clades and isolates in the BBB need further studies. We have also not included antiretrovirals, which per se may contribute to BBB dysfunction despite keeping most drugs from entering the CNS [95], increasing the in vivo complexity. The complex pharmacological interactions between current HIV suppressive treatments and substance use are a separate chapter.

Using this controlled system with two different cerebrovascular cell lines, our results confirm the value of CB2R-mediated recovery of the BBB in the context of inflammation and suggest a contribution of a cAMP threshold underlying protective cellular events. While it is possible that upregulation of CB2R caused by inflammation enhances a signaling threshold that can shift the endothelial cell response via CB2R towards functional improvement, our results show that endothelial cells stimulated with HIV-latent U1-derived conditioned media had a similar decrease in levels cAMP, recovered by the selective agonist, and more likely by THC. However, the overall higher expression of CB2R in hCMEC/D3 compared to HBMEC/ci18, leading to beneficial conditions, may contribute to an increased strength of activation of this signaling pathway, thereby promoting vascular health. The results suggest that HU-308, the CB2R agonist, is invisible at a fully-tone-active receptor and only becomes functionally relevant once that tone is pathologically suppressed, satisfying a receptor-reserve/threshold model.

The present data also provide evidence that p38 MAPK phosphorylation is engaged downstream of CB2R activation by CBD/THC in both cerebrovascular endothelial cell lines, though with markedly different kinetics and magnitude. In hCMEC/D3 cells, CBD/THC induced a rapid and robust increase in pp38, peaking at 5–10 min and resolving to near-baseline by 60 min, consistent with the transient MAPK activation profile characteristic of GPCR-coupled signaling. In HBMEC/ci18 cells, the pp38 response was substantially attenuated, reaching a lower peak at 5 min and declining progressively thereafter. Crucially, pre-incubation with the CB2R inverse agonist AM630 suppressed the pp38 response approximately five-fold in hCMEC/D3 and effectively abolished it in HBMEC/ci18, directly implicating CB2R in the upstream regulation of this pathway. CB2R couples primarily to Gi/o proteins, whose Gβγ subunits are well-established activators of p38 MAPK through a PI3Kγ–Rac1–MKK3/6 cascade [96,97,98]. The rapid kinetics of the pp38 response are consistent with this Gβγ-mediated route, which operates independently of transcriptional intermediates and is therefore faster than stress- or cytokine-driven p38 activation [99]. The cell-line difference in pp38 magnitude is moreover consistent with the differential CB2R expression and cAMP response capacity described above: lower receptor availability in HBMEC/ci18 would be expected to yield reduced Gβγ release and proportionally attenuated downstream MAPK signaling. While CBD and THC are pharmacologically pleiotropic and may engage additional p38-activating targets including TRPV1 and GPR55, the AM630-mediated suppression of pp38 establishes CB2R as a necessary upstream component of this response in both cell lines. Whether p38 activation in this context is mediated through canonical Gαi/o–Gβγ signaling, β-arrestin recruitment following receptor desensitization, or a combination of both, remains to be formally resolved and would benefit from selective Gβγ inhibition or PI3Kγ blockade in future studies.

It is important to acknowledge that THC and CBD are pharmacologically pleiotropic compounds acting across multiple receptor targets including CB1R, CB2R, GPR55, TRPV1, and others, and that their divergent effects from HU-308 cannot be specifically attributed to CB2R signaling thresholds without additional receptor-specific experiments or effects on beta-arrestin, which remain to be examined. Yet, the reduction of CBD/THC-induced forskolin-sensitive cAMP levels and downstream phosphor p38, suggest that CB2R GPCR capacity plays an important role in endothelial health in the context of HIV. The AM630 data establishes CB2R as necessary but not necessarily sufficient or exclusive.

The data highlights that endothelial heterogeneity may be an important component of relevant vascular outcomes. The two cell lines studied here, hCMEC/D3 and HBMEC/ci18, are both derived from human cerebral microvasculature, yet they differ in their immortalization strategies [100,101], passage history, transcriptomic profiles (not shown), and baseline signaling capacities, as evidenced throughout this study by their divergent CB2R expression, cAMP thresholds, GPCR pathway signaling, and functional responses to cannabinoids. Regional, developmental, and individual differences in endothelial identity have been increasingly recognized as determinants of BBB permeability and drug responses [102,103,104,105]. Clinically, this heterogeneity implies that the beneficial or aggravating effects of cannabinoids on the BBB may vary across individuals depending on their cerebrovascular endothelial phenotype, a consideration relevant to PWH, who show inter-individual variability in neurocognitive outcomes as well as in cannabis-associated inflammatory markers.

Overall, these findings inform the molecular basis that is critical for the development of novel therapeutic strategies that can improve BBB health as an underlying mechanism in NCI. Experimental approaches in humanized animal models may serve as a critical bridge to test whether CB2R expression levels and cAMP thresholds in cerebrovascular endothelium can indeed predict differential cannabinoid responses in vivo, and for investigating if pharmacological strategies to elevate baseline CB2R expression or cAMP tone could confer protection in a susceptible endothelial background. Longitudinal clinical studies in PWH that capture both cannabinoid use patterns and vascular biomarkers are an additional priority, especially based on our prior observational work on VCAM-1, ICAM-1, and uPAR as BBB integrity biomarkers in this population [56,57].

## Data Availability

The datasets used and/or analyzed during the current study are available from the corresponding author on reasonable request.

## Abbreviations

The following abbreviations are used in this manuscript:

BBB	Blood-Brain Barrier
BrdU	5-Bromo-2′-deoxy-Uridine
BSA	Bovine Serum Albumin
cAMP	cyclic adenosine 3,5 monophosphate
CB1R	Cannabinoid Receptor-1
CB2R	Cannabinoid Receptor-2
CBD	Cannabidiol
CBRs	Cannabinoid Receptors
CMEC	cerebral microvascular endothelial cells
CNS	Central Nervous System
CSF	Cerebrospinal Fluid
CST3	Cystatin C
CVD	Cardiovascular Disease
DAPI	4′,6-diamidino-2-phenylindole
DMEM	Dulbecco’s Modified Eagle Medium
DMSO	Dimethyl sulphoxide
ECS	Endocannabinoid system
EGF	Endothelial Growth factor
ELISA	Enzyme-linked Immunosorbent Assay
FAAH	Fatty Acid Amide Hydrolase
GAPDH	Glyceraldehyde-3-phosphate dehydrogenase
GFAP	Glial Fibrillary Acidic Protein
GPR55	G-protein receptor 55
GS	Glutamine Synthetase
HASTR	human astrocytes
HBMEC	human brain microvascular endothelial cells
HBPC	human brain pericytes
HBSS	Hank’s balanced salt solution
HIV	Human Immunodeficiency Virus
ICAM-1	intercellular adhesion molecule-1
IGFBP4	insulin-like growth factor binding protein-4
MAGL	Monoacylglycerol lipase
MAPK	mitogen-activated protein kinase
MPO	myeloperoxidase
NCI	Neurocognitive Impairment
PBS	calcium- and magnesium-free phosphate buffered saline
PET	clear polyester
PI3K	activate phosphoinositide 3-kinase
PIGF	Placental Growth Factor
PMA	Phorbol Myristate Acetate
PWH	Persons living with HIV
RAGE	receptor for advanced glycation end products
RNA	Ribonucleic Acid
SAA	Serum Amyloid A
SPP1	Osteopontin
THC	Δ9-tetrahydrocannabinol
TJ	tight junctions
TNFa	Tumor Necrosis Factor-alpha
uPAR	urokinase activator plasminogen receptor
VCAM-1	vascular cell adhesion molecule-1
VEGF	Vascular Endothelial Growth Factor
ZO-1	Zonula Occludens-1
